# Effects of innovative reinforced concrete slit shaft configuration on seismic performance of elevated water tanks

**DOI:** 10.1038/s41598-024-56851-3

**Published:** 2024-03-13

**Authors:** Filip Gurkalo, Chaofan He, Konstantinos Poutos, Na He

**Affiliations:** 1 School of Civil Engineering, Henan Polytechnic University, Jiaozuo, Henan China; 2 Faculty of Engineering, Computing and the Environment, Kingston University, London, UK

**Keywords:** Elevated water tank, Nonlinear, SAP2000, Finite element analysis, Earthquake, Time history analysis, Engineering, Civil engineering

## Abstract

Elevated water tanks are considered crucial infrastructure due to their significant role in supporting essential services. A strong ground motion may result in a failure or significant damage to a reinforced concrete shaft of an elevated water tank because hysteric energy dissipation is limited to the formation of plastic hinges at the base of the shaft, while the nonlinear properties of the rest of the shaft remain underutilised. The innovative system of assembling RC shafts for elevated water tanks using a slit wall technique was developed to enhance energy dissipation along with the shaft height by introducing slit zones. The comparative nonlinear dynamic analysis between three-dimensional models of elevated water tanks with different shaft diameters and heights was conducted using SAP2000 software. The results of elevated water tanks with slit and solid reinforced concrete shafts were compared. The research findings showed that during a seismic event, the slit zones increased the ductility of the shaft, reduced stress concentration in the lower part of the shaft, and provided uniform stress distribution throughout the shaft's height. The effect of the innovative system is especially noticeable in the elevated water tanks with tall and slender shafts.

## Introduction

In numerous regions across the globe, households lack a consistent provision of potable water and must depend on scheduled water collection to meet their needs. Elevated water tanks (EWT) are commonly constructed to function as a vital reservoir and a means of generating pressure for a water distribution network. They play a crucial role in regions where the availability or reliability of electricity for pumping operations is restricted. Moreover, EWTs are widely recognised as essential infrastructure and are expected to continue functioning during and after an earthquake^1,2^. Damage to these structures can limit their functionality in meeting the needs for drinking water and fire extinguishment, especially after strong earthquakes that considerably boost these demands.

The structural integrity of EWTs is mainly compromised by damage to their supporting framework^3^. Generally, the supporting structure of the elevated water tanks can be classified as either a reinforced concrete frame, steel frame, masonry shaft, or a reinforced concrete shaft. In this study, the term “EWT” specifically refers to the water tanks mounted on the reinforced concrete (RC) shafts. A key part of such a system is the hollow reinforced concrete shaft through which loads are transferred to the foundation.

The structural characteristics of the RC shaft resemble those of the hollow cylindrical columns since the shaft wall thickness (usually 150–400 mm) is significantly smaller than its diameter (usually 5–20 m). Unlike most other structures, elevated water tanks undergo varying gravity loads (empty and full conditions) and low redundancy due to the absence of load redistribution paths^4^. Also, monolithic elevated water tanks have relatively high strength and stiffness but lack ductile characteristics. Ductile behaviour in the RC shafts is achieved by yielding reinforcement at the shaft base and forming plastic hinges^5^. Due to low redundancy and poor ductility in thin reinforced concrete shafts, considerable damage in the hollow RC shaft during a strong seismic event may result in total collapse or affect the functionality of EWT^6^. The literature provides evidence of the inadequate structural performance of reinforced concrete shafts in EWTs during past seismic events^7–11^. The extent of damage ranged from minor cracks in the shafts to the complete collapse of the entire structure.

The total energy transferred to the structure can be dissipated through two mechanisms: damping energy and hysteretic energy. The only amount of dissipated energy due to the inelastic deformation is considered to damage the structure subjected to an earthquake. Based on this criterion, the collapse of a structure can be described as an inability to dissipate hysteretic energy through inelastic deformation^12^. Furthermore, a number of scholars utilised hysteretic energy as a parameter for seismic design^13,14^. In RC structures, hysteretic energy is an appropriate parameter due to the representation of cumulative nonlinear responses such as cracking and plastic hinging of the ductile members.

Generally, two categories of methods are used to make EWT resistant to earthquakes: conventional and nonconventional. A conventional method refers to the approach of enhancing the design capacity and stiffness of the structure^15^. Load-bearing capacity and flexural stiffness can be enhanced by increasing RC shaft thickness and reinforcing materials to reduce the danger of damage or structural failure of an EWT. However, this arrangement would lead to an increased seismic impact due to the higher stiffness of the shaft.

On the other hand, an alternative approach involves mitigating seismic demand rather than enhancing strength through the implementation of base isolation devices^16–18^. The primary purpose of base isolation systems is to separate the superstructure from the substructure by inserting a flexible layer, such as rubber bearings or sliders, at the foundation level. Base isolators are designed to absorb ground motion and elevate the structure above the ground, maintaining a nearly fixed position during an earthquake. During an earthquake, the kinetic energy of the earthquake is absorbed into heat energy by base isolators. That transfers the structure into a lower frequency range, where the seismic energy acting on the structure exceeds that of resonance. However, when it comes to EWTs with tall shafts, the possibility of maintaining a fixed position is uncertain because of the concentration of mass at the top^19^.

In comparison to the conventional and nonconventional methods, this study presents an innovative system of assembling RC shafts for elevated water tanks using a slit wall technique. In the case of high-intensity earthquakes, flexible support systems are preferred as they can receive large deformations. On the other hand, stiff support systems should be considered for frequent low-intensity earthquakes or wind action because they prevent large displacements. In other words, the earthquake response of the structure can be reduced by modifying the shaft design.

The dissipation of the hysteric energy in the RC shaft of EWT is comparable to the shear wall system that generally occurs through the concentrated plastic hinge formation at the lower part of the wall, and the ductility resources of the remaining wall remain unexploited.

Numerous investigations have been conducted to enhance the ductility of shear walls exposed to seismic forces by diminishing the energy concentration at the base of the shear wall and redistributing it across the entire height. In the 1970s, Muto proposed a more advanced version of the shear wall known as the slit shear wall^20^. This innovation aimed to enhance the performance of shear walls in resisting lateral forces. The presence of slits in the wall and connectors between parts of the wall resulted in an observable enhancement in ductility and seismic energy dissipation. Subsequent investigations by other researchers demonstrated that slit shear walls had enhanced ductility and reduced stiffness compared to conventional shear walls^21^.

Kwan et al.^22^ improved a model of a slit wall. Reinforced concrete beams connected two parts of slit walls though out all heights of a slit wall, and connectors formed a dissipative zone. The comparison between solid and slit walls was made, and results showed the efficiency of the slit wall: the displacements and story drift decreased by 20% as well as overall ductility of the structure was improved. It was concluded that seismic performance depended on the yielding resistance of the connections. Therefore, the rational design of connectors was of great importance.

Seismic damage evaluation of reinforced concrete buildings with slit walls was investigated by Baietu et al.^23^. It was determined that the presence of slit walls in the building enhances its ability to dissipate energy. During a high-intensity seismic event, if the connections fail, the stiffness and strength of the slit walls decrease, allowing more seismic force to be transmitted into the frames. This results in the entire building exhibiting ductile behaviour.

Labafzadeh et al. studied inelastic dynamic analysis on various shear walls with different opening arrangements^24^. Results showed that using a rational arrangement of openings in the shear wall led to the dispersing of the hysteric energy across the height of the wall and employed both flexural and shear ductility capacity of the system at the base and around the openings, respectively. In addition, the responses of the slit shear wall, such as base shear, base moment, top story displacement, and the average value of inter-story drift along the height, were reduced compared to the solid wall.

Kheroddin et al.^25^ introduced the optimal placement of coupling elements of RC shear walls. It was found that the utilisation of viscoelastic coupling dampers results in a decrease in the lateral stiffness of the structure and a shift in the natural period beyond the prominent periods of typical earthquakes, hence improving the seismic performance of the structure.

The behaviour of coupled shear walls was studied by Nofal et al.^26^. The investigation was conducted to analyse the impact of coupling beam characteristics on the nonlinear behaviour of the system consisting of coupled shear walls. A 10-story linked shear wall system was comprehensively analysed using finite element simulations. The findings suggested that a span-to-depth ratio serves as a critical threshold for the behaviour of the coupling beams. Ordinary flexure mainly influences the behaviour when the ratio is greater than two. Conversely, deep beam behaviour predominantly determines the behaviour when the ratio is less than two. The findings indicated that the coupling beam’s width does not substantially influence the reaction of the linked shear wall. Furthermore, it was determined that the excessive diagonal reinforcement in the coupling beam could substantially impact the behaviour of the coupled shear walls. As a result, a maximum limit for the diagonal reinforcement was established.

The innovative system of assembling RC shafts for elevated water tanks using a slit wall technique was developed by Gurkalo et al.^27^. In that study, the researcher attempted to determine the optimal width of slits to minimise stress concentration at the shaft base and uniformly distribute stresses along the shaft height, which could lead to a decreased demand ductility capacity at the base. The capacity spectrum analysis was employed to evaluate the performance of EWT. Finite Element Analysis (FEA) results demonstrated that the width of the slits in the RC shaft had a substantial impact on both the failure mode and stiffness of the water tower. The most optimal response was achieved with a slit width equal or less than 100 mm.

**Figure 1:**
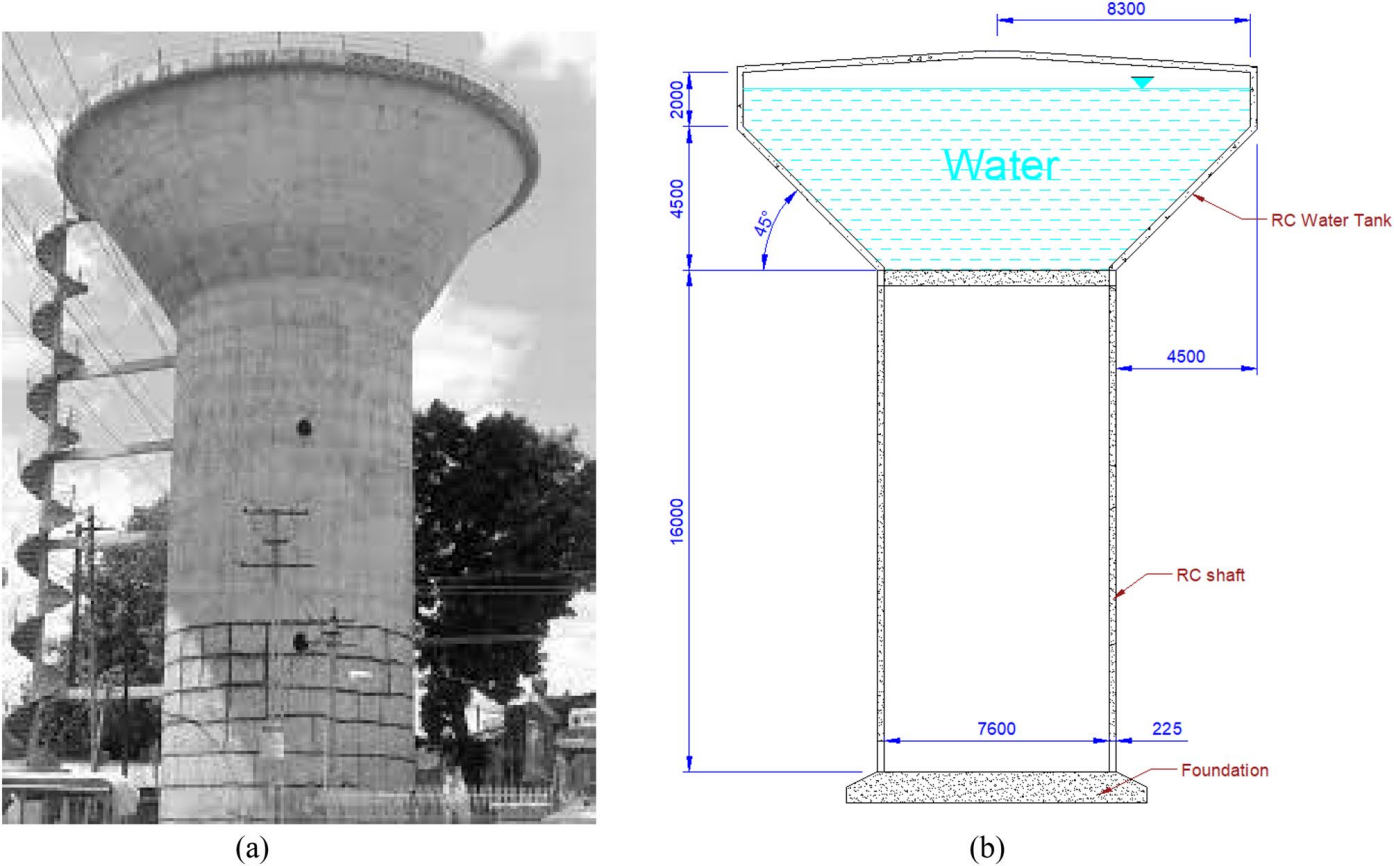
(**a**) Anjar Nagar Palika elevated water tank^28^. (**b**) Simplified model of an Anjar Nagar Palika EWT.

The aim of this study was to evaluate the impact of slits in the RC shafts of EWTs on the seismic response. The key variables under investigation were the diameter and height of the shaft. The time history approach was utilised in order to determine the dynamic nonlinear response in the slit EWTs and compare the results with those obtained from conventional RC solid shaft EWTs. The Anjar Nagar Palika EWT geometry was considered a benchmark for the solid model. Figure 1a shows the damaged Anjar Nagar Palika EWT during the Bhuj earthquake on January 26th, 2001, and Fig. 1b shows its simplified model^28^.

## Methodology

### Study models of solid and slit elevated water tanks

The primary objective of this study is to investigate the impact of shaft height and diameter on the seismic response of the proposed slit EWTs. The construction of slit shafts involved the incorporation of four evenly spaced slits positioned at 90-degree intervals, spanning the entire shaft height. Additionally, the RC shaft quarters were interconnected by a foundation located at the base of the shaft and a ring beam situated at the uppermost part of the shaft. In addition, a series of interconnected beams, spaced at intervals of 5 m, were included in the model to connect the foundation with the ring beam. The width of the slots was determined to be 50 mm, as recommended by Gurkalo^27^. The split shaft versions possessed identical overall dimensions and material qualities to the solid shaft. The C20/25 concrete and reinforcement were configured in two layers, with a spacing of 275 mm, in both the transverse and longitudinal directions, as observed in the original Anjar Nagar Palika EWT investigated by Rai^28^. All characteristics of solid and slit EWTs are presented in Table 1.

**Table 1:** Characteristics of elevated water tanks used in this study.

	Solid shaft	Slit shaft
Internal shaft diameter	6.6 m, 7.6 m or 8.6 m	6.6 m, 7.6 m or 8.6 m
Shaft height	11 m, 16 m, 21 m or 26 m	11 m, 16 m, 21 m or 26 m
Shaft wall thickness	225 mm	225 mm
Water tank diameter	16.6 m	16.6 m
Water tank height	6.5 m	6.5 m
Water tank wall thickness	250 mm	225 mm
Water tank floor thickness	300 mm	300 mm
Capacity of the water tank	1000 kL	1000 kL
Ring beam	500 mm × 225 mm	500 mm × 225 mm
Coupled beam	N/A	350 mm × 225 mm
Slit width	N/A	50 mm
Coupled beam location	N/A	Every 5 m
Concrete	C20/25	C20/25
Shaft reinforcement	2 layers ${\varnothing }10$ every 275 mm for both transverse and longitudinal reinforcement	2 layers ${\varnothing }10$ every 275 mm for both transverse and longitudinal reinforcement
Beam longitudinal reinforcement	2 bars ${\varnothing }25$—compression 2 bars ${\varnothing }25$—tension	2 bars ${\varnothing }25$—compression 2 bars ${\varnothing }25$—tension
Beam traverse reinforcement	2 bars ${\varnothing }10\text{ every }150\text{ mm}$	2 bars ${\varnothing }10\text{ every }150\text{ mm}$

**Figure 2:**
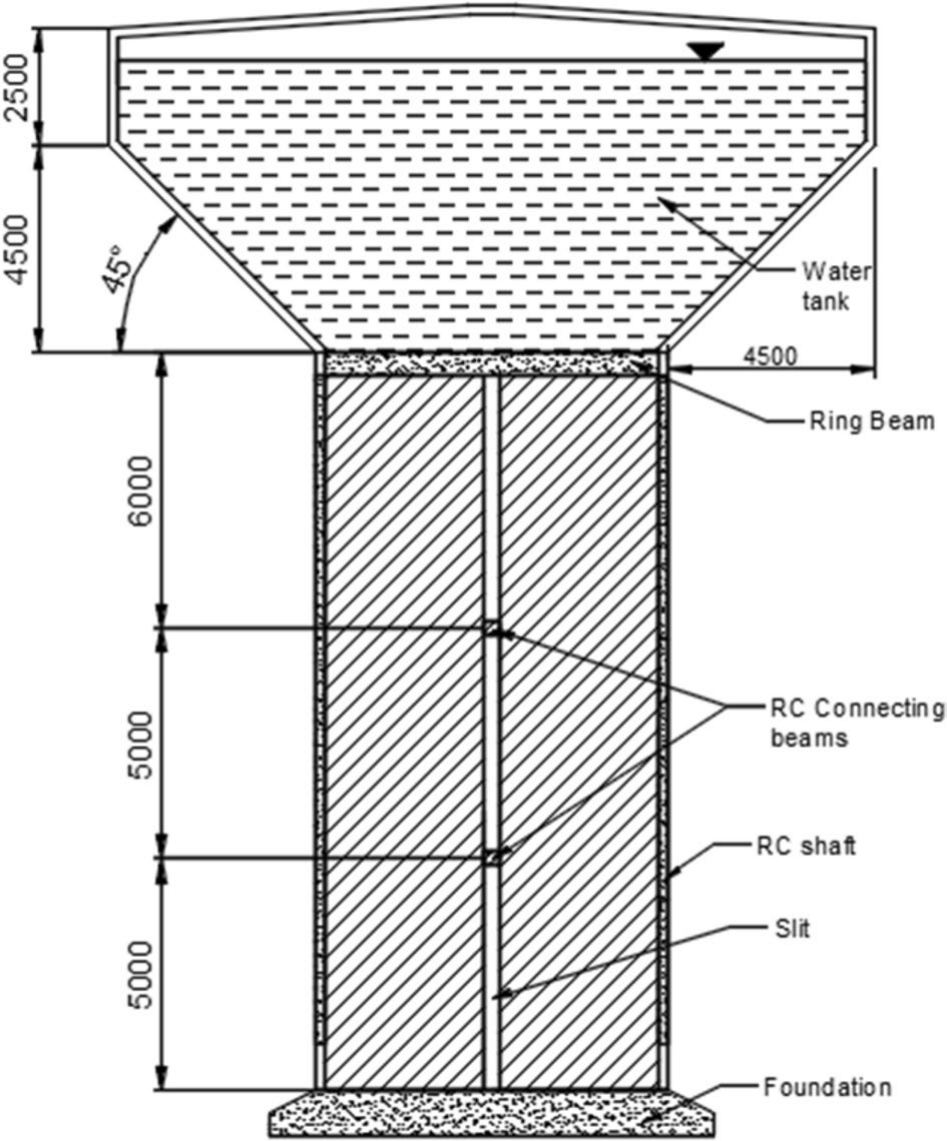
Proposed Slit-17-7.6 model.

This study involved 12 finite element (FE) models with different shaft heights and diameters, including 6 EWT with solid shafts and 6 EWT with slit shafts. Table 2 presents the FE model identification number (ID) assignment to each FE model. The initial phrase denotes the EWT, which can be either a solid or a slit structure. The subsequent term signifies the vertical height of the shaft, measured in metres. Lastly, the final term symbolises the diameter of the EWT's shaft, measured in metres. Hence, the FE model denoted as ID Slit-16-7.6 indicates the FE model of an EWT with a slit shaft of 16 m in height and 7.6 m in diameter (Fig. 2).

**Table 2:** FE model ID of selected elevated water tanks.

Shaft height (m)	Solid EWT			Slit EWT		
	Shaft diameter (m)					
	6.6 m	7.6 m	8.6 m	6.6 m	7.6 m	8.6 m
11	N/A	Solid-11-7.6	N/A	N/A	Slit-11-7.6	N/A
16	Solid-16-6.6	Solid-16-7.6	Solid-16-8.6	Slit-16-6.6	Slit-16-7.6	Slit-16-8.6
21	N/A	Solid-21-7.6	N/A	N/A	Slit-21-7.6	N/A
26	N/A	Solid-26-7.6	N/A	N/A	Slit-26-7.6	N/A

### Finite element analytical models

**Figure 3:**
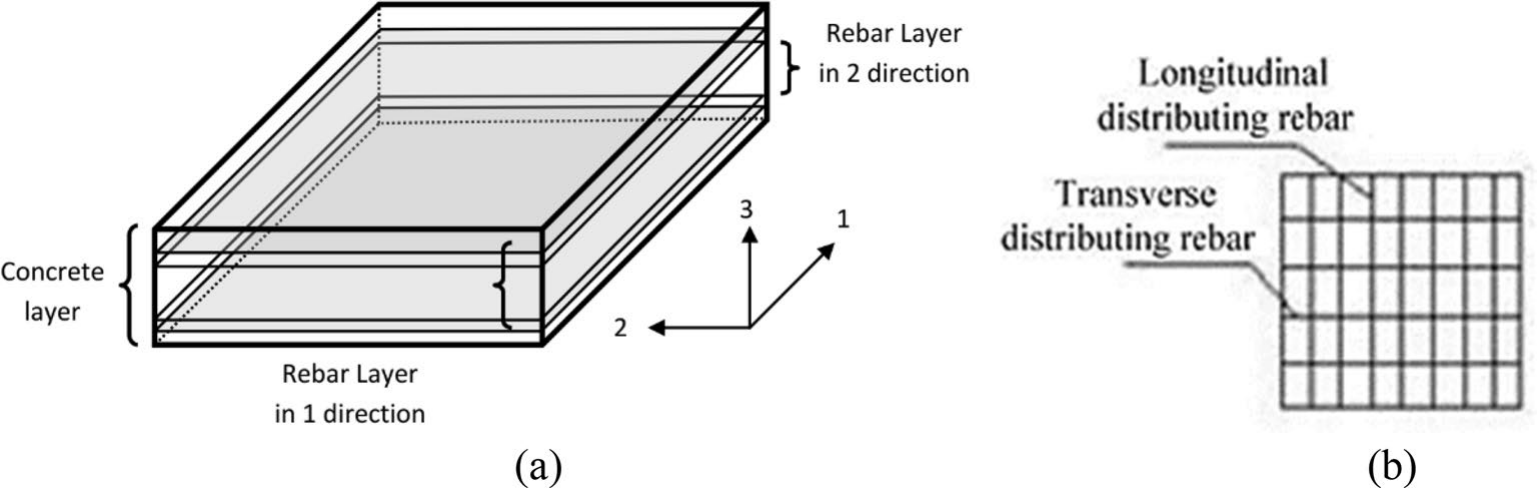
Layered four-node quadrilateral shell element^30^.

This study incorporated both geometric nonlinearity and material nonlinearity in the modelling of an RC shaft. The wall thickness of the RC shaft in EWT is considerably smaller than the shaft height and diameter. As a result, FE models of both solid and slit shafts were modelled using four-node quadrilateral shell-layered elements, as described in the SAP2000 manual^29^ (Fig. 3a).

According to Wilson^30^, using shell elements was effective in conducting analyses of reinforced concrete (RC) shafts interconnected with beams. A shell element, frequently employed in design software, possesses six degrees of freedom at each node and an additional degree of freedom for in-plane rotation. Thus, shell elements are compatible with three-dimensional and beam-type elements.

The layered shell element under consideration is founded upon the concepts of composite material mechanics. It can replicate the interconnected in-plane/out-plane bending and the interconnected in-plane bending-shear nonlinear behaviours of an RC shaft with the individually treated concrete and steel components^31^. The chosen constitutive model for the rebar was the ideal elastoplastic model. The rebar in the longitudinal and transverse orientations were designed separately (Fig. 3b).

This study considered the effects of P-Δ and large displacements associated with geometrical nonlinearity. The P-Δ effect is a significant factor in nonlinear modelling and analysis concerning the displacements relative to the ends of structural members. The force of gravity affects the structural response when there is a considerable lateral displacement. Consequently, the P-Δ effect may play a role in the reduction of lateral resistance, the accumulation of residual deformations, and the occurrence of dynamic instability^32^.

The coupled beams were simulated using a frame element defined in SAP2000. The modelling of the nonlinear behaviour of beams involved the incorporation of a stiff plastic spring at the anticipated yielding region. The region between the two stiff plastic springs exhibited complete elasticity, with any inelastic deformation considered confined to these springs. The development of the nonlinear model for beams was founded upon using the plastic hinge concept and incorporating a bilinear moment-rotation connection^33^. The SAP2000 software includes fibre-plastic hinges that define the plastic zones at the beam ends. The hinge entailed the procedure of partitioning the segment into several longitudinal fibres. The axial stress–strain relationship was determined for each fibre in the cross section by utilising the material nonlinear stress–strain curve. The axial force–deformation and biaxial moment-rotation equations are obtained by aggregating the behaviour of all the fibres within the cross-section and multiplying them by the hinge length. According to Park and Pauley^34^, the accepted hinge length was determined to be 0.5 times the height of the beam.

Generally, RCEWT can be categorised into three substructures: the tank, shaft, and foundation. The primary objective of this work is to investigate the nonlinear behaviour of the RC shaft. Consequently, some simplifications were implemented to represent the remaining two substructures.

**Figure 4:**
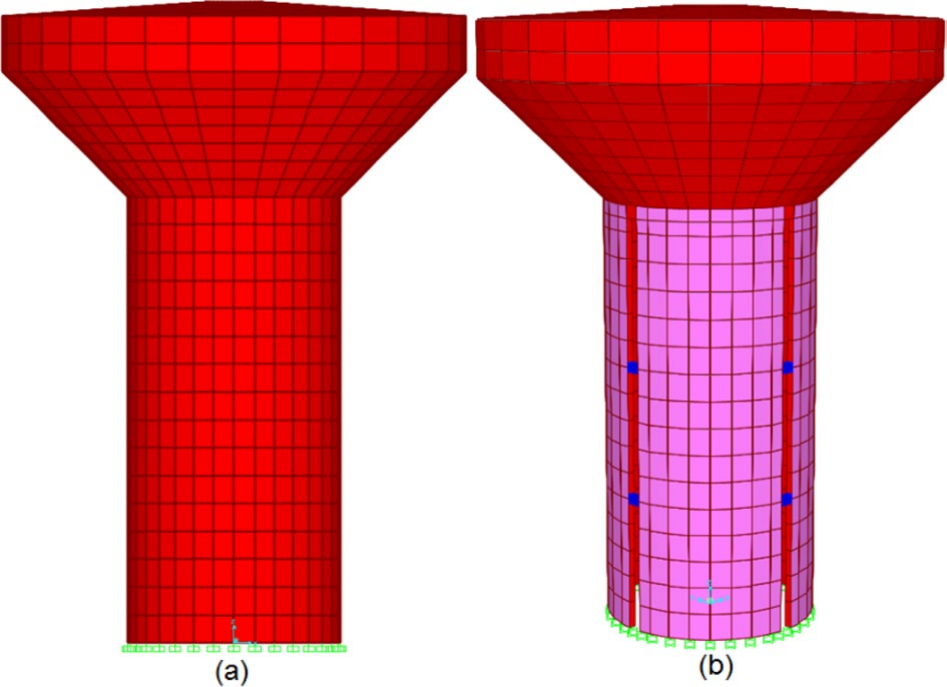
Finite element model of (**a**) solid elevated water tank (**b**) slit elevated water tank.

The water tank was excluded from the scope of this study. Thus, linear, thin shell elements were implemented for its modelling. It was assumed that the foundation possessed a high degree of rigidity, and the shaft was affixed at the same elevation as the foundation. The application of boundary conditions involved the constraints of all degrees of freedom at the base level of the reinforced concrete shaft. Figure 4 shows FE models of EWTs with solid and slit shafts.

Stress–strain behaviour for concrete and reinforcing bars is essential in the inelastic FEA of reinforced concrete structures. Separate stress–strain curves for concrete and steel were used in this study due to their significant material behaviour disparity. The stress–strain model proposed by Mander^35^ was chosen to represent concrete behaviour, whereas the model proposed by Holzer^36^ was selected to represent steel rebar.

**Figure 5:**
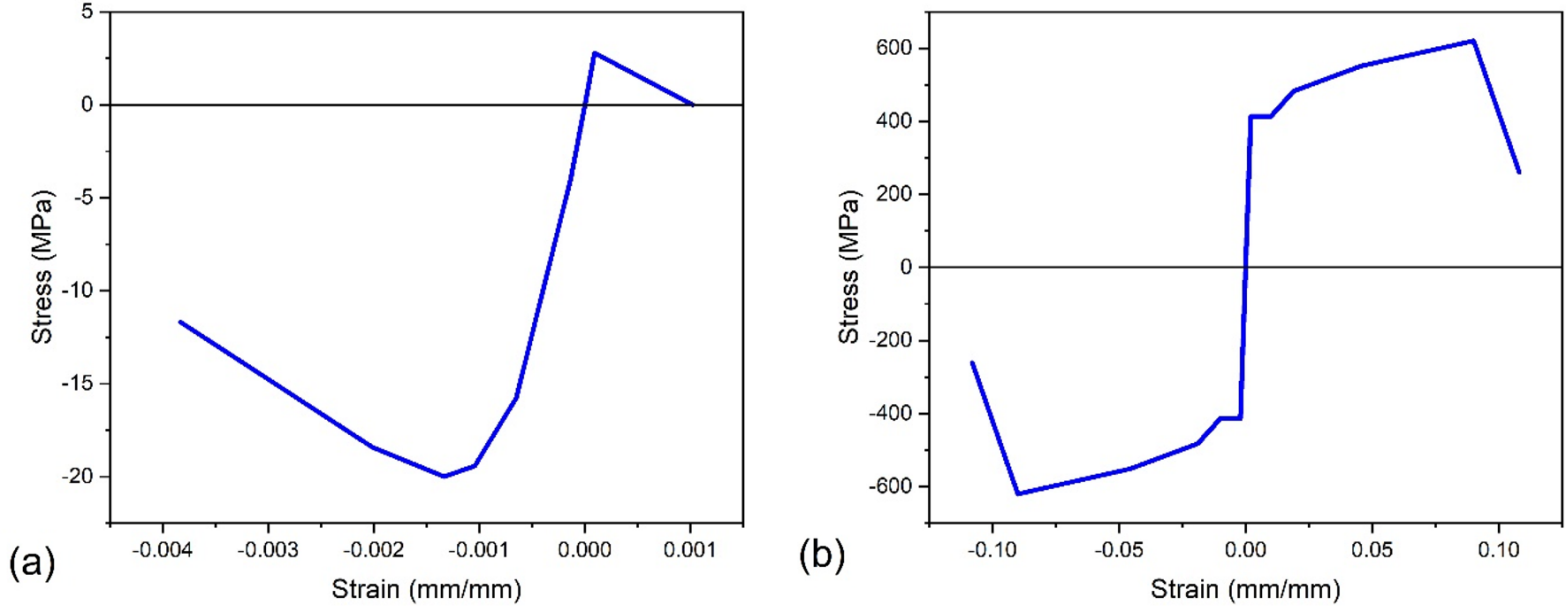
(**a**) The stress–strain curve for C20/25 concrete^35^. (**b**) The stress–strain curve for rebar^36^.

The mechanical properties of the concrete material with a grade of C20/25 were specified as follows: the compressive strength, denoted as ${f}_{c}{\prime}$, was determined to be 20 N/mm^2^, while the tensile strength, denoted as *f*_*t*_, was determined to be 2.79 N/mm^2^. Young’s modulus was assumed to have a value of 30 kN/mm^2^, and the Poisson’s ratio was taken as 0.2. Additionally, the strain at compressive strength, denoted as ${\varepsilon }_{c}{\prime}$, was determined to be 0.00133, while the ultimate strain, denoted as ${\varepsilon }_{u}$, was determined to be 0.00383. Figure 5a displays the stress–strain curve representing C20/25 concrete.

In order to enhance structural integrity, the mechanical properties of steel included the yield strength (*f*_*y*_) and ultimate strength (*f*_*u*_), which were determined to be 14 N/mm^2^ and 620 N/mm^2^, respectively. Additionally, the yielding strain (${\varepsilon }_{y}$) and ultimate strain (${\varepsilon }_{u}$) were identified as 0.00207 and 0.09, respectively. Young's modulus value of 200 N/mm^2^ and Poisson’s ratio value of 0.29 were used in this study. Figure 5b shows the rebar’s stress–strain relationship.

**Figure 6:**
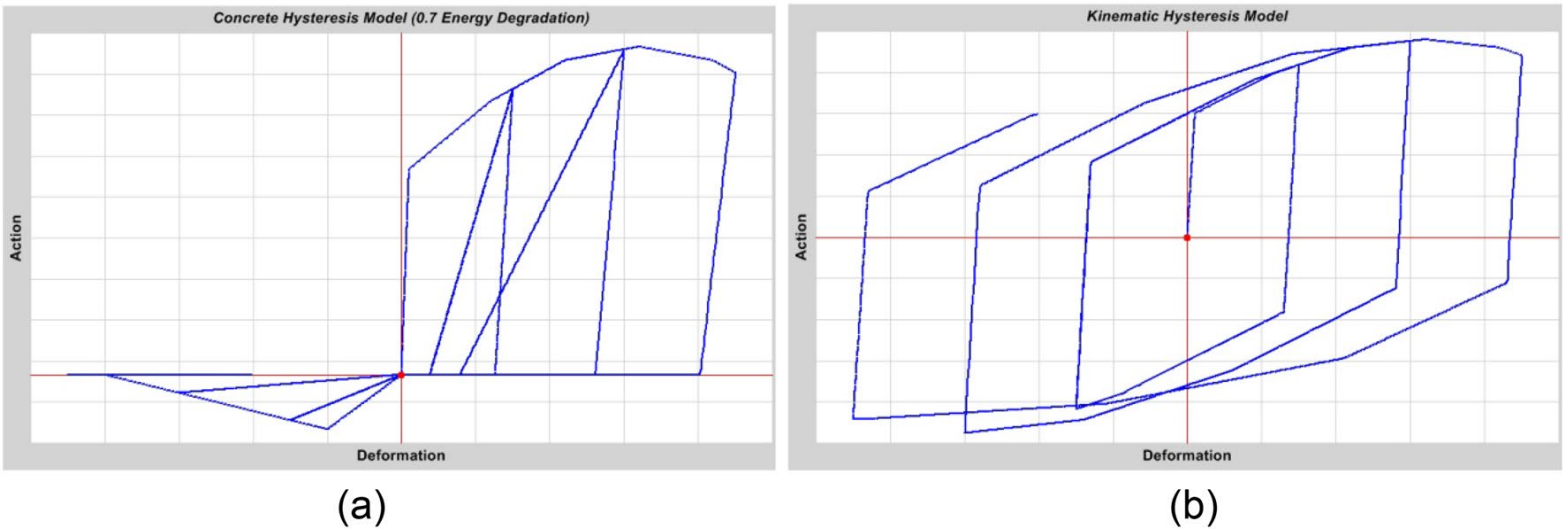
(**a**) Concrete hysteresis model under increasing cyclic load with compression as positive. (**b**) Steel hysteresis model under increasing cyclic load^29^.

Hysteresis refers to energy dissipation resulting from deformation, specifically displacement, in contrast to viscosity, which involves energy dissipation associated with deformation rate or velocity. Various hysteresis models can be employed to characterise the behaviour of diverse materials. In a general sense, there exist differences between materials in terms of the amount of energy they dissipate during a particular cycle of deformation and how their dissipation behaviour evolves with increasing distortion. The hysteresis models employed in this work, which pertain to concrete and steel materials, are shown in Fig. 6.

**Figure 7:**
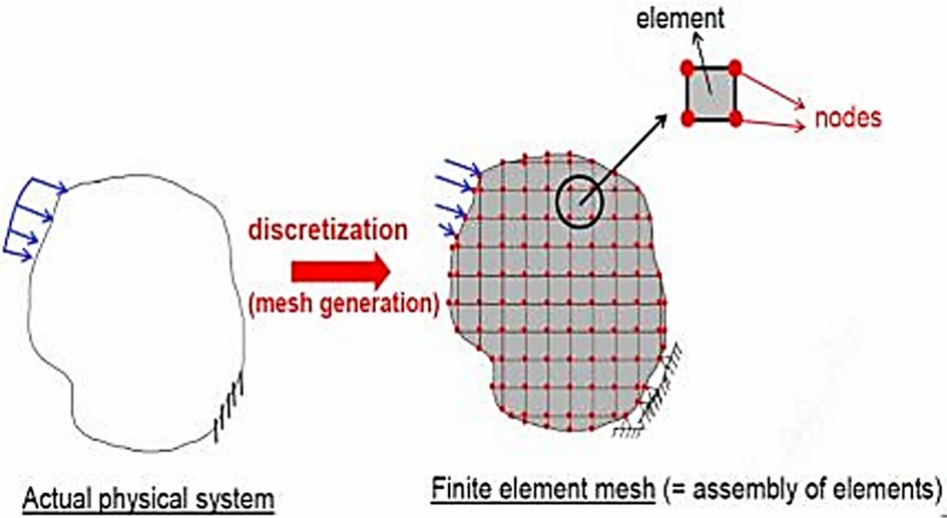
Finite element discretization.

Finite element models offer a means of analysing structures with nonlinear RC shaft behaviour, dividing a physical region into a mesh of finite elements, as seen in Fig. 7. The method employed in this methodology consists of utilising material constitutive laws and the assumption of a deformation pattern through the implementation of approximate shape functions. Solutions are subsequently derived using the displacements and forces at discrete places along a finite element, commonly called nodes. The finite element model produces results that accurately depict the system under consideration by employing a sufficiently small mesh size in conjunction with an adequate deformation pattern (shape functions) and constitutive material models.

The analysis of the RC shaft was conducted using the finite element method, specifically employing a two-dimensional plane stress shell model. Hence, in order to accurately capture the behaviour of shear walls, it was necessary to engage mesh discretisation for modelling purposes. The shaft and tank were simulated using a refined mesh consisting of quadrilateral shell elements to ensure precise outcomes.

### Simplified model of water

A water-retaining structure, such as a water tank, can provide a significant complexity to finite element modelling due to the interaction between the fluid and the structure. The primary dynamic phenomenon of liquid sloshing is the horizontal oscillations of the liquid waves within a container. Using complex finite element models in engineering practice is not rational, so engineers often opt for simplified models that yield precise outcomes. The mechanical approach involves inducing horizontal oscillation in the tank, which causes the convective component to move, imitating the liquid sloshing phenomenon. However, the vertical oscillation of the tank does not exert any discernible impact on the liquid.

**Figure 8:**
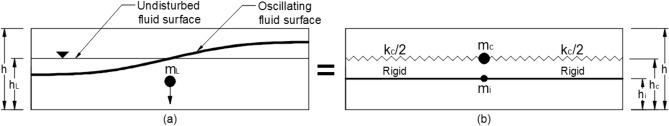
(**a**) Fluid motion in a water tank, (**b**) mechanical model of liquid.

A simplified model for the analysis procedure suggested by Housner^37^ was employed in this study. When a liquid-filled tank is subjected to seismic excitation, the forces acting on the tank wall can be classified into two separate components: impulsive and convective. Both the impulsive and convective components can be represented by an equivalent mechanical model, as shown in Fig. 8. It was accepted that the impulsive mass (*m*_*i*_) is firmly affixed to the tank walls at the height (*h*_*i*_) above the tank base. In contrast, the convective mass (*m*_*c*_) is connected to the tank walls through springs positioned at the height (*h*_*c*_) above the tank base.

The procedure of Housner’s model of an elevated tank subjected to horizontal dynamic load can be realized by impulsive and convective masses can be determined as:

1 $${m}_{i}={m}_{f}\frac{{\text{tanh}}\left(1.74\frac{R}{h}\right)}{\left(1.74\frac{R}{h}\right)}$$

2 $${m}_{c}={m}_{f} 0.318\frac{R}{h}{\text{tanh}}\left(1.84\frac{R}{h}\right)$$

where, m, R and h are the total fluid mass, radius of the vessel and height of the fluid in the vessel, respectively; *h*_*i*_ and* h*_*c*_, symbolizing the heights of the impulsive and convective masses from the vessel base, that can be determined by the following equations:

3 $${h}_{i}=\frac{3}{8}h$$

4 $${h}_{c}=\left[1-\frac{{\text{cosh}}\left(1.84\frac{R}{h}\right)-1}{1.84\frac{h}{R}{\text{sinh}}\left(1.84\frac{R}{h}\right)}\right]h$$

The stiffness of a spring, denoted as *k*_*c*_, can be calculated by:

5 $${k}_{c}={{\text{m}}}_{{\text{c}}}\frac{g}{R}1.84{\text{tanh}}\left(1.84\frac{h}{R}\right)$$

According to the guidelines outlined in Eurocode 8^38^, it is suggested that an approximate estimation for the impulsive and convective masses of axisymmetric tanks, which are not cylindrical, can be derived by considering an equivalent cylindrical tank. This comparable tank should have the same diameter for its free surface and a water depth that yields an equal volume of water for both the original and equivalent tanks. The scenario of a filled tank was unanimously acknowledged as the most severe example due to the highest levels of base shear and top lateral displacement in EWTs during seismic events.

**Figure 9:**
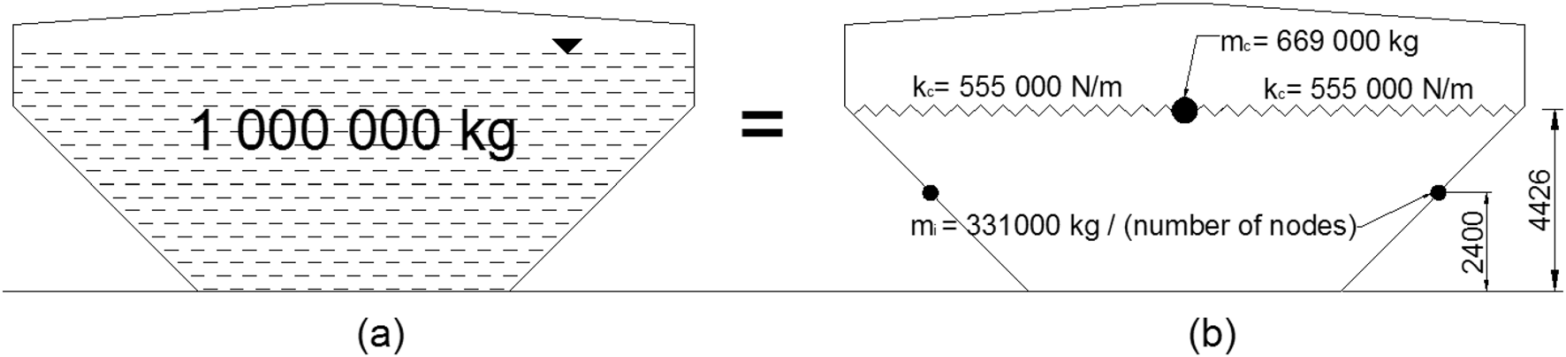
(**a**) Water tank of proposed models, (**b**) equivalent two-mass model.

The capacity of the water tank was 1000 m^3^. It was thought that the conversion factors 1 m^3^=1000 l = 1000 kg were valid, leading to the supposition that the total mass contained within the water tank was 1,000,000 kg. Figure 9 shows the two-mass water model contained within the water tank utilised for this study.

### Damping

When doing dynamic analysis of a structure using the direct method, it is possible to incorporate Rayleigh damping to consider the damping characteristics of the structure. In this scenario, a damping matrix [C] is constructed by combining the mass [M] and stiffness [K] matrices by the multiplication of mass and stiffness Rayleigh proportional coefficients α and β, respectively, as follows^39^:

6 $$[C]=\alpha [M]+\beta [K]$$

The precise values of α and β are typically not directly known but rather derived from the modal damping ratios ${\zeta }_{n}$, representing the ratio of actual damping to critical damping for a specific mode of vibration n. Let ${\omega }_{n}$ be the natural circular frequency of mode n. The variables α and β are subject to a relation:

7 $${\zeta }_{n}=\frac{\alpha }{2{\omega }_{n}}+\frac{\beta {\omega }_{n}}{2}$$

**Figure 10:**
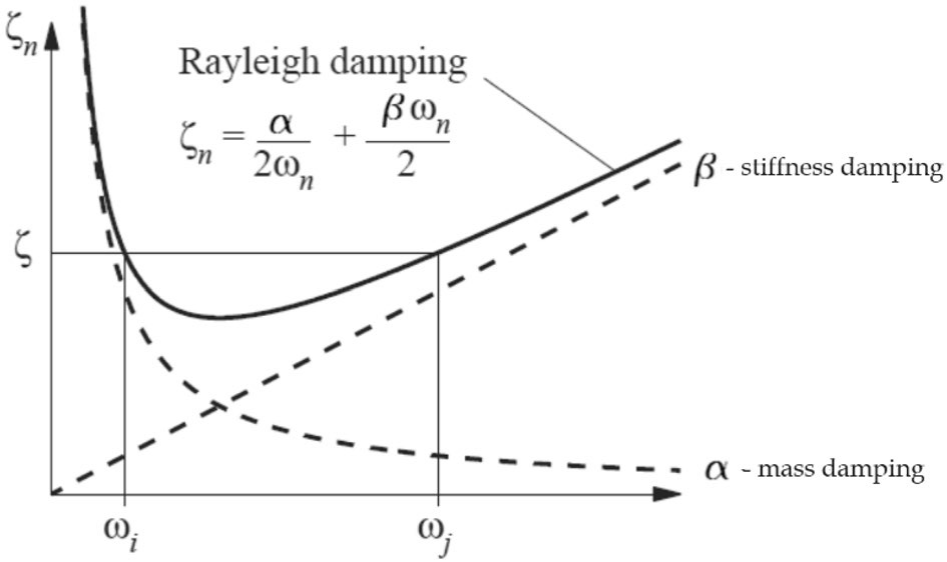
Rayleigh damping^40^.

The application of Rayleigh damping results in a comprehensive curve that effectively aligns with modal damping values at specific natural frequency points. Therefore, when a structure exhibits one or two prominent frequencies, using Rayleigh damping can accurately approximate the dynamic response of the structure. By considering the same damping ratio for both *i*th and *j*th modes of the structure, the graphical representation of Rayleigh damping in relation to the modal damping ratio (ζ) and natural cyclic frequency (ω) is shown in Fig. 10. The choice of proper *i*th and *j*th modes is crucial, i.e., the specified modes should significantly contribute to the structures’ response^35^.

As suggested by EC-8, the damping ratios of 0.5 and 5 percent are assigned for the convective and impulsive components, respectively. Furthermore, the stiffness proportional damping equivalent to 5 percent of critical damping is assumed as structural damping^40^.

### Nonlinear time history analysis

When structures experience substantial dynamic loads, such as seismic excitation, it is necessary to evaluate their nonlinear response. The optimal approach to include the impacts of nonlinearity in dynamic analysis is utilising a time domain solution, commonly referred to as time history analysis. This method is considered the most precise approach for determining the actual reaction of buildings under intense ground motions. The methodology employed in this approach is founded upon a step-by-step integration. According to Yu^41^, the step-by-step method involves dividing the loading and response history into intervals. The reaction at each time increment is determined based on the original state. Moreover, it is assumed that the structural qualities remain constant, and the equation of motion maintains elasticity over each time increment Δ*t*.

SAP2000 finite element software was used to perform direct integration implicit time-history analysis, employing Newmark’s average acceleration method^42^ in conjunction with the Newton–Raphson approach^43^. This choice was made due to the lack of high-frequency noise in the models.

**Figure 11:**
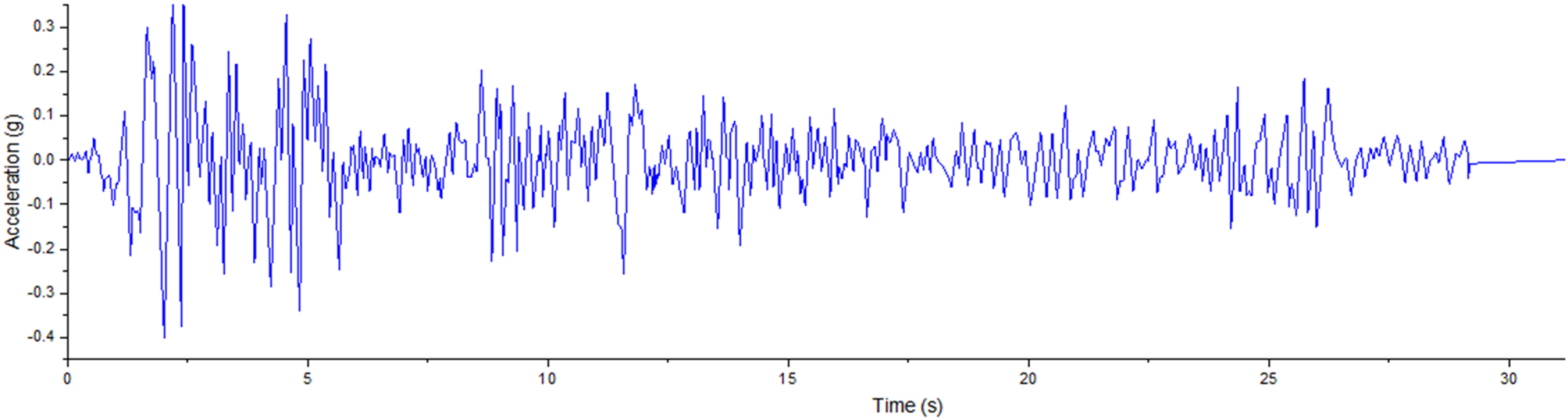
1940 El-Centro ground motion, horizontal component^44^.

The ground acceleration used for the time history study was derived from the horizontal component of the 1940 El Centro earthquake^44^, as shown in Fig. 11. The chosen EWT models underwent unidirectional horizontal seismic excitation. For the time history analysis, a time step of 0.005 s was employed for integration.

## Results and discussion

### Modal analysis

The modal analyses were performed on the three-dimensional finite element models.

**Figure 12:**
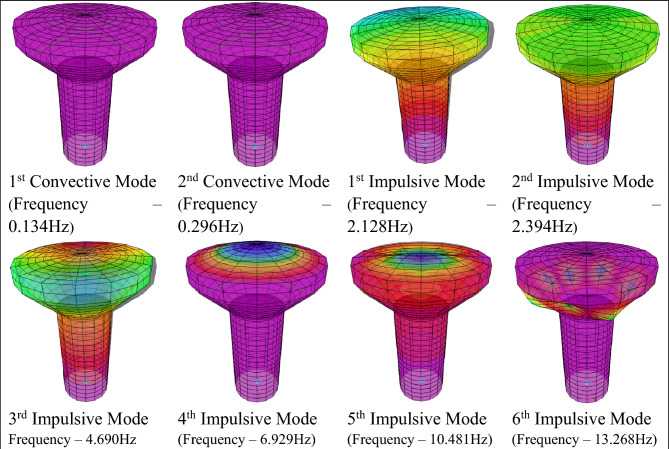
Solid-16-7.6 model mode shapes.

Based on Eurocode 8, it is necessary to employ a sufficient number of modes to ensure that the combined effective masses of these modes account for at least 90% of the total mass of the structure. Figure 12 shows the first eight mode shapes of the Solid-16-7.6 model, including 2 conventional modes and 6 impulsive modes that combined effective modal masses participation ratios in both horizontal directions were 95%. The mode shapes and mass participation ratios are similar for all models.

Modes with similar fundamental periods represented the same mode in two perpendicular directions (X and Y). Each of these modes was deviated from the X or Y axis by a small angle (1st and 2nd impulsive modes). As a result, the mode shapes and other modal properties remained similar for the first two convective and the first two impulsive modes.

The fundamental impulsive mode exhibited translational characteristics. This particular mode can be categorised as the cosθ type mode, wherein the tank's cross-section maintains a circular shape. During the primary impulsive mode, the EWT exhibited characteristics similar to those of a vertical cantilever beam.

For higher impulsive modes, the top part of the vessel experiences more pronounced deformation compared to the rest of the tank. Moslemi et al.^45^ explained this phenomenon by the higher stiffness attributes associated with the conical part compared to the cylindrical part.

The fundamental period is a term that includes both the geometric and dynamic response characteristics of structures. Identifying the fundamental period of a water-retaining structure exposed to horizontal seismic forces is of most significance, as resonance effects are responsible for most tank failures during seismic events. The fundamental periods in this study were influenced by several key factors, namely the kind of shaft (solid or slit), the shaft's height, and the shaft's diameter.

Identifying the fundamental impulsive periods was based on determining the modes with the highest participation factors in the horizontal direction. The effective masses associated with the fundamental modes showed much higher values than those of the other modes. This suggests that the system's response was mainly influenced by the modes related to horizontal excitations. Table 3 represents the fundamental periods for all models.

**Table 3:** Fundamental periods of finite element (FE) models.

FE model ID	Fundamental period (s)	FE model ID	Fundamental period (s)
Solid-11-7.6	0.350	Slit-11-7.6	0.376
Solid-16-6.6	0.526	Slit-16-6.6	0.587
Solid-16-7.6	0.418	Slit-16-7.6	0.493
Solid-16-8.6	0.383	Slit-16-8.6	0.425
Solid-21-7.6	0.558	Slit-21-7.6	0.633
Solid-26-7.6	0.698	Slit-26-7.6	0.790

**Figure 13:**
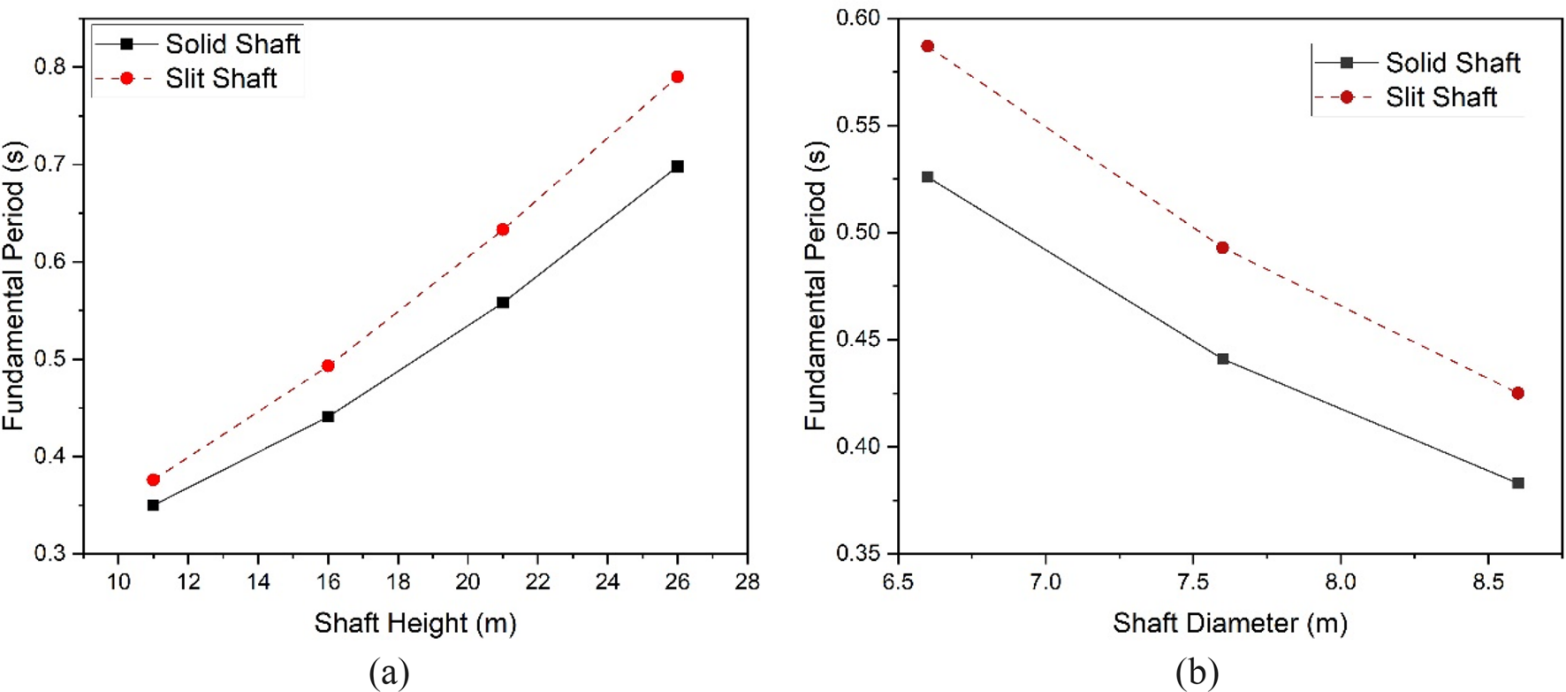
Fundamental periods of solid and slit EWTs with different (**a**) shaft heights, (**b**) shaft diameters.

The elongation of the fundamental periods was observed when the shaft height increased (Fig. 13a), and the diameter decreased (Fig. 13b) in both the solid and slit models. The observed outcome was anticipated due to the alteration in shaft height, which led to increased flexibility and a reduction in diameter. Moreover, the augmentation in shaft height and the decrease in shaft diameter have a more pronounced impact on the fundamental periods observed in solid and split EWTs.

There was a difference of 0.026 s in the natural periods between the Solid-11-7.6 and Slit-11-7.6 models. In contrast, there was a difference of 0.092 s in the natural period between the Solid-26-7.6 and Slit-26-7.6 models. The difference in fundamental periods between solid and slit EWTs, with shaft heights of 16 m, was observed to be 0.061 s, 0.052 s, and 0.042 s for models featuring shaft diameters of 6.6 m, 7.6 m, and 8.6 m, respectively.

### Nonlinear time history analysis

#### Effect of the shaft height

To assess the impact of shaft height on the dynamic characteristics of solid and slit EWTs, models with a consistent diameter of 7.6 m were selected. These models encompassed four distinct shaft heights: 11 m, 16 m, 21 m, and 26 m. The proposed time history analysis method was employed to perform the comparative study. The solid and slit models were exposed to a horizontal seismic excitation from the El-Centro direction. The resulting values of shear force and flexural moment response at the base of the shafts and the lateral displacement at the top of the EWTs were calculated and then compared. Base shear, base moment, and top lateral displacement responses of the solid and slit FE models subjected to El-Centro horizontal excitation are shown in Supplementary Material.

Table 4 summarises the maximum time history response values for the solid and slit EWT models. The bold numbers indicate the percentage change in response values of slit shaft models compared to solid shaft models. Positive values indicate an increase, while negative values indicate a decrease.

**Table 4:** Time history response values for 7.6 m shaft diameter models subjected to El-Centro excitation.

	Shaft height							
	11 m		16 m		21 m		26 m	
	Solid	Slit	Solid	Slit	Solid	Slit	Solid	Slit
Base shear (MN)	4.8	4.6 **(− 3.2%)**	4.5	3.9 **(− 11.9%)**	3.6	3.1 **(− 13.7%)**	3.1	2.7 **(− 10.5%)**
Base moment (MNm)	76.6	72.4 **(− 5.6%)**	89.1	78.3 **(− 12.1%)**	89.3	79.5 **(− 10.9%)**	86.4	76.1 **(− 10.9%)**
Top lateral displacement (mm)	19.7	24.6 **(+ 24.8%)**	35.9	44.8 **(+ 24.7%)**	53.6	58.3 **(+ 8.8%)**	61.3	61.9 **(+ 1.0%)**

**Figure 14:**
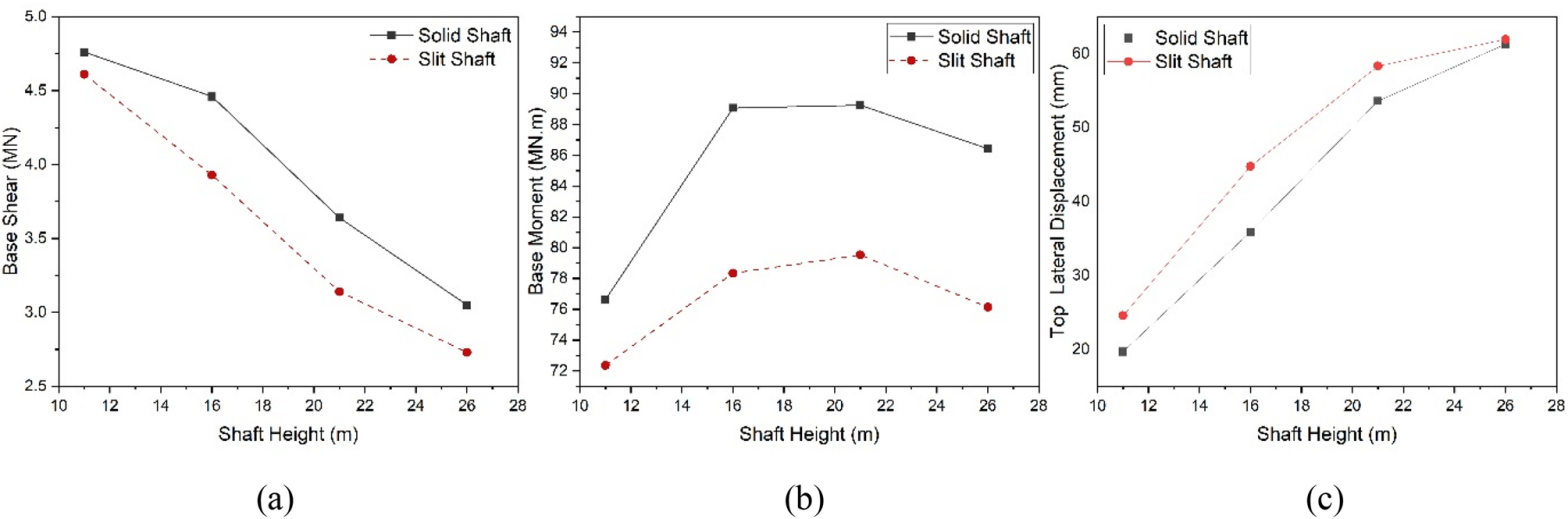
Maximum response values of solid and slit EWT models with different shaft heights: (**a**) base shear, (**b**) base moment, (**c**) top lateral displacement.

The analysis of the response variations for both the solid and slit EWTs revealed a distinct reduction in stiffness in the time-history response of the slit shaft models across all the models examined. The analysis of the base shear reveals that the base shear in the slit EWTs showed a reduction of 3%, 12%, 14%, and 11% for models with shaft heights of 11 m, 16 m, 21 m, and 26 m, respectively, when compared to the solid models (Fig. 14a). The analysis of the base moment revealed a 9%, 12%, 11%, and 12% reduction for models with shaft heights of 11 m, 16 m, 21 m, and 26 m, respectively, compared to solid models (Fig. 14b).

The data indicates that the maximum base moment for both solid and slit EWTs occurred at the heights of the shafts (16 and 21 m), after which it exhibited a decline. The observed behaviour can be attributed to variations in shaft stiffness, resulting in distinct water behaviours that subsequently impact the centre of mass within the water tank. Nevertheless, as shown in Fig. 14c, a distinct pattern was noted regarding the lateral displacement reaction at the uppermost section of the tank. In the context of slits, it is often preferred to utilise shafts with more flexibility than those with higher stiffness. The analysis of the highest lateral displacement revealed that the displacement in slit EWTs exhibited an increase of 25%, 25%, 9%, and 1% for models with shaft heights of 11 m, 16 m, 21 m, and 26 m, respectively, as compared to solid versions.

#### Effect of the shaft diameter

In order to perform a comparative analysis on the impact of shaft diameter on slit and solid EWTs, models with identical shaft heights of 16 m were utilised. These models featured three distinct shaft diameters: 6.6 m, 7.6 m, and 8.6 m. A time history analysis was performed to assess their behaviour under horizontal seismic excitation from the El Centro seismic record, acquiring their respective time history responses. Base shear, base moment, and top lateral displacement responses of the solid and slit FE models subjected to El-Centro horizontal excitation are shown in Supplementary Material.

Table 5 summarises the maximum base shear, flexural moment, and top lateral displacement values for the solid and slit EWT models. The figures in bold indicate the percentage change, either positive or negative, compared to the solid shaft models. The relationship between shaft diameter and the reduction in base shear and base moment is evident, as both parameters increase with an increase in shaft diameter for both solid and slit models. Conversely, an increase in diameter leads to a rise in top lateral displacement.

**Table 5:** Time history response values for models with 16 m shaft height subjected to El-Centro excitation.

	Shaft diameter					
	6.6 m		7.6 m		8.6 m	
	Solid	Slit	Solid	Slit	Solid	Slit
Base shear (MN)	3.7	3.1 **(− 16.9%)**	4.5	3.9 **(− 11.9%)**	5.0	4.9 **(− 2.4%)**
Base moment (MNm)	81.9	72.8 **(− 11.1%)**	89.1	78.3 **(− 12.1%)**	98.5	94.6 **(− 3.9%)**
Top lateral displacement (mm)	48.6	55.2 **(+ 13.6%)**	35.9	44.8 **(+ 24.7%)**	24.2	36.6 **(+ 51.3%)**

**Figure 15:**
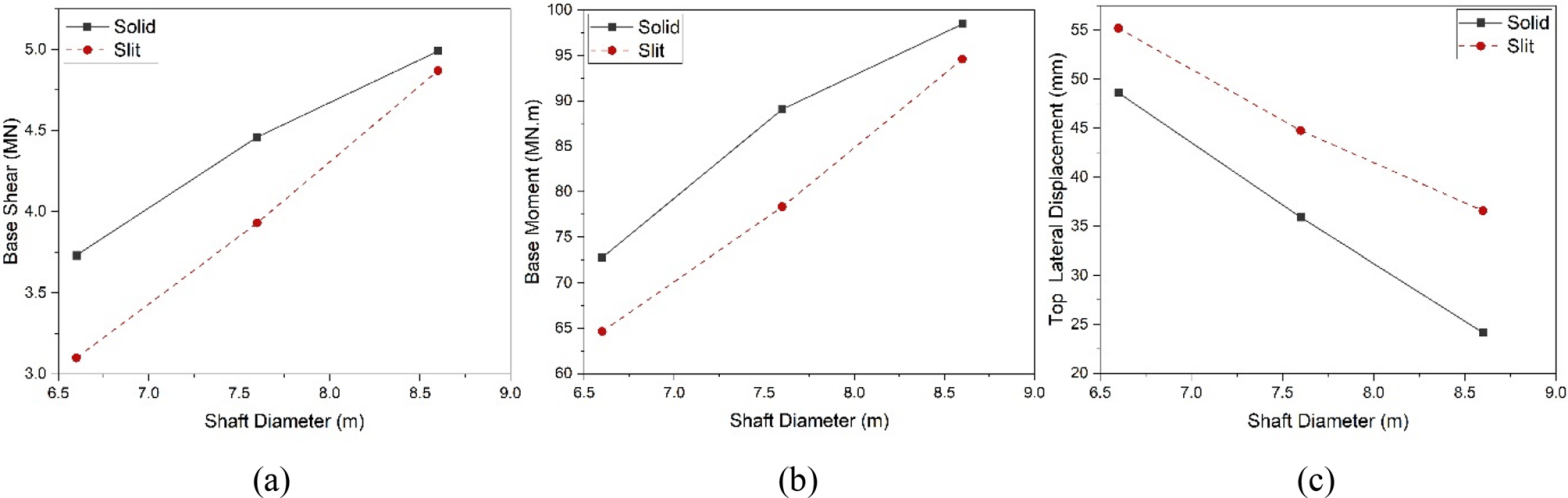
Maximum response values of solid and slit EWT models with different shaft diameters: (**a**) base shear, (**b**) base moment, (**c**) top lateral displacement.

A notable augmentation in the disparity of the base shear and base moment was seen when comparing solid and slit models when the shaft diameter was reduced. The analysis of the base shear revealed that the base shear in the slit EWTs showed a reduction of 17%, 12%, and 2% for models with shaft diameters of 6.6 m, 7.6 m, and 8.6 m, respectively, when compared to the solid EWTs (Fig. 15a). In addition, it was observed that the base moment showed a reduction of 11%, 12%, and 4% in the slit models with shaft diameters of 6.6 m, 7.6 m, and 8.6 m, respectively, compared to the solid models (Fig. 15b).

However, as shown in Fig. 15c, a distinct pattern was noted in the lateral displacement reaction at the uppermost section of the tank. The magnitude of lateral top displacement exhibits a negative correlation with the diameter of the shaft. The analysis of the highest lateral displacement revealed that the displacement in EWTs with slits rose by 14%, 25%, and 51% for models with shaft diameters of 6.6 m, 7.6 m, and 8.6 m, respectively, compared to solid versions.

### Stress distribution in the RC shafts

Studying the locations of the principal stress concentrations in concrete enhances comprehension of the structure's vulnerable areas and its reaction to seismic loads. It was assumed that the highest stress occurred at the maximum lateral displacement at the top of the tank. Cracking was assumed to occur in concrete areas when the maximum principal stress (tension stress) reached the ultimate tensile concrete strength of ${f}_{t}{\prime}=2.785\, \text{N/mm}^{2}$. After this point, the tension load was taken mainly by reinforcement, and displacement became more noticeable in the cracked area. On the other hand, when the minimum principal stress (compression stress) reached the ultimate compressive concrete strength of ${f}_{c}{\prime}=-20\, \text{N/mm}^{2}$, concrete began to crash, leading to the failure of an elevated water tank.

**Figure 16:**
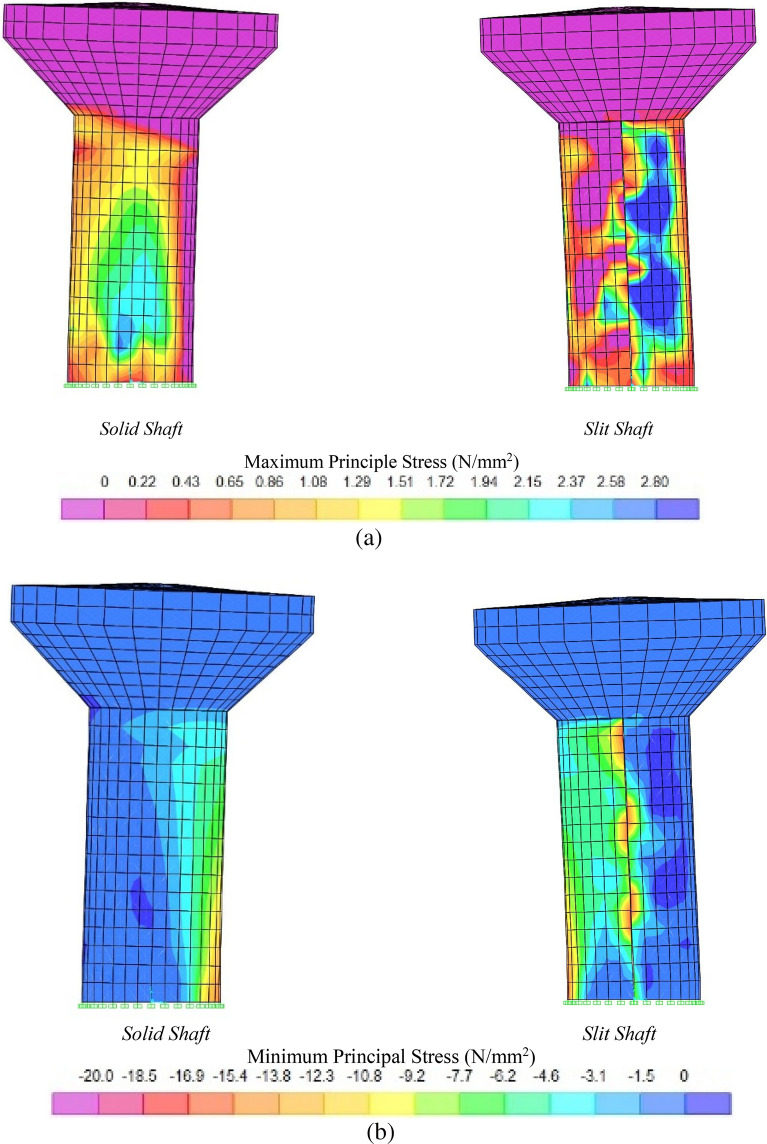
Contours of the maximum principal stress (**a**) and minimum principal stress (**b**) distribution in concrete in solid and slit RC shafts at peak top lateral displacement subjected to El Centro earthquake.

Figure 16 shows the contours of the principal stress distribution in solid and slit RC shafts during the peak top lateral displacement caused by the El Centro earthquake. Based on the observed patterns, it can be stated that EWTs with solid shafts experience the highest maximum principal stress, primarily in the lowest one-third of the shaft. In contrast, the remaining portion of the shaft remains underutilised. Moreover, the highest magnitude of the minimum principal stress was observed at the locations of the shaft's base, which were oriented perpendicular to the earthquake's direction. Additionally, this stress did not propagate uniformly along the entire shaft length.

Cracking propagation can be analysed by considering the distribution of stress. In the case of solid models, the cracking formation began with flexural tension cracks at the bottom of the shaft. Ductile behaviour in the shaft occurred by yielding flexural reinforcement at the shaft base. Then, cracks extended rapidly parallel to the lateral load direction. The cracks observed near the base of the shaft can be referred to as base-shear cracks, which were caused by the combined influence of flexure and shear. Finally, significant crack propagation occurred throughout the lower portion of the shaft. By forming the flexural plastic hinge, large relative displacements take place at the top of the EWTs, having a small contribution to dissipating energy.

Conversely, slit RCEWTs have a consistent distribution of the maximum and minimum stresses throughout the vertical axis of the shaft. The shaft showed the highest maximum principal stress in shaft walls oriented parallel to the ground motion, leading to web-shear cracking. The minimum principle stress concentrations were detected in the connection zones with coupling beams. The observed stress distribution pattern exhibited greater desirability due to the absence of stress concentration at the base of the reinforced concrete (RC) shaft. Instead, stresses were uniformly distributed throughout the whole shaft, facilitating energy dissipation over its entire length.

**Figure 17:**
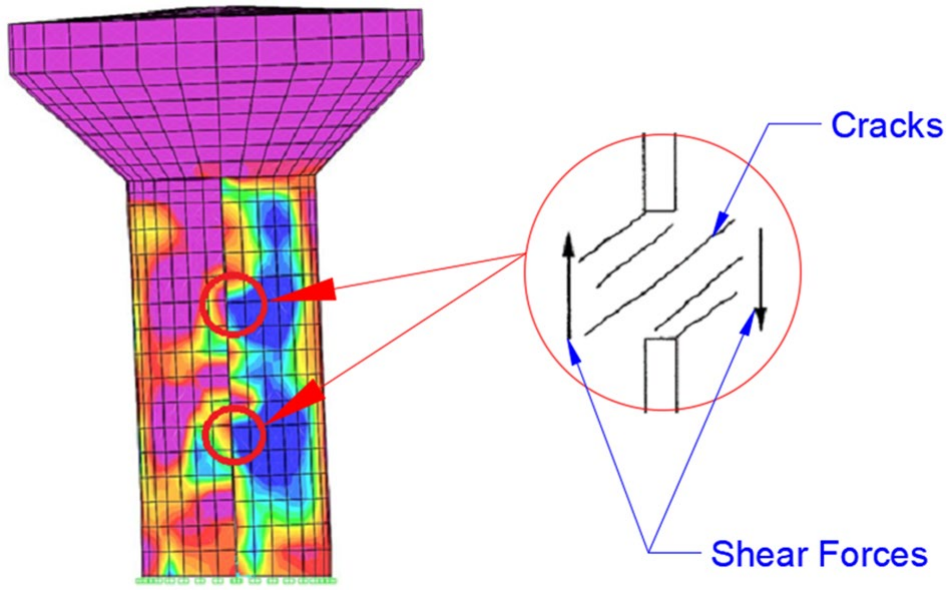
A typical crack pattern of a connection beam region.

The stress pattern for the slit models differs from the solid models. First, yielding and formation of plastic hinges occurred at the connections of shaft piers with connection beams mainly due to the produced shear force in the connection region (Fig. 17). The shear force resulted from the flexural deformation of the shaft piers. The progression of cracks around connection beams was initiated near the upper connections of the shaft and progressed both upwards and downwards. Once the region around the coupling beams began to crack, the coupling action started to degrade, and the lateral forces, once resisted by coupling frame action, were distributed to the shaft piers. The cracks located around the connections can be classified as web-shear cracks. Then, cracking of the base part of the shaft had begun, and the cracks propagated across the shaft. The ring beam remained elastic the longest.

It was identified that the slit shaft had a better hysteresis energy dissipation capacity that could prevent severe damage to the shaft base. The energy dissipation mechanism is different for slit shafts and solid shafts. The slit shaft dissipates hysteresis energy via cracks extended on the entire surface of the shaft and plastic hinge formation in the connection beams. However, the solid shaft dissipates seismic energy only by cracks at the base of the shaft.

Many researchers stated that plastic hinges in connection beams and around connections should appear first for better energy dissipation and distribution of cracks along the whole structure^46^. The depth and reinforcing ratio of the connecting beams directly impact the failure mode and ductility of a slit EWT. Designing the connection beams with good ductility and yield strength is recommended to achieve optimal overall performance, ensuring that they do not fail significantly earlier than the wall. In order to ensure that the beams do not yield in advance but yield before the wall does. Kwan A.K.H.^22^ suggested that the beams should have a yield strength ranging from 50 to 100% of the shear load they could experience while remaining in the elastic state when the shaft yields. For best performance, the shear connections should maintain their load carrying and energy dissipation capacities until the whole structure fails.

## Conclusions

The following conclusions were derived from the findings of this study:

- The presence of slits in reinforced concrete shafts enhances their ductility and reduces the stiffness, resulting in an elongation of the fundamental periods.
- The elongation of fundamental periods increased when shaft height increased, and diameter decreased in both solid and slit models. However, the difference in fundamental periods between models with solid and slit shafts increased with an increase in shaft height and diameter. The difference in fundamental periods between solid and slit models with a shaft diameter of 7.6 m increased from 0.026 s for models with 11 m shaft height to 0.092 s for models with 26 m shaft height. Moreover, the difference in fundamental periods between solid and slit models with shaft height of 16 m increased from 0.061 s for models with 6.6 m shaft diameter to 0.075 s for models with 8.6 m shaft diameter.
- The difference in base shear and base moment between solid and slit EWTs with the same shaft diameter of 7.6 m as the shaft height increased became more pronounced, and the difference in top lateral displacement became less noticeable. Namely, the difference increased from 3.2 to 10.5% in base shear and from 5.6 to 10.9% in the base moment and decreased from 24.8 to 1.0% in top lateral displacement when comparing EWTs with shaft heights of 11 m and 26 m, respectively.
- The difference in base shear and base moment between solid and slit EWTs with the same shaft height of 16 m as the shaft diameter increased became less obvious, and the difference in top lateral displacement became more prominent. Specifically, the difference decreased from 16.9 to 2.4% in base shear and from 11.1 to 3.9% in the base moment and increased from 13.6 to 51.3% in top lateral displacement when comparing EWTs with shaft diameters of 11 m and 26 m, respectively.
- The efficacy of the slit EWTs mainly depended on the geometric characteristics of the RC shaft. The presence of slits in RC shafts significantly impacted the seismic behaviour of tall and slender EWTs, such as the model with shaft height and diameter of 26 m and 7.6 m, respectively. However, the impact of slits in RC shafts in short and broad EWTs was negligible to seismic response, such as the model with shaft height and diameter of 11 m and 8.6 m, respectively.
- The elevated water tank models with slit shafts effectively mitigated stress concentration at the base of the shaft, showing uniform tension and compression stress distribution along the shaft height compared to solid models with stress concentration at the lowest 1/3 part of the shaft.

### Supplementary Information

Supplementary Figures.

## Data Availability

All data generated or analysed during this study are included in this published article and its Supplementary Material file.
