# Supplementary material for: Effects of innovative reinforced concrete slit shaft configuration on seismic performance of elevated water tanks
Source: Sci Rep. 2024 Mar 13;14:6113. doi: 10.1038/s41598-024-56851-3 (PMC10937947; doi:10.1038/s41598-024-56851-3)
Supplement: Supplementary file 1 — Supplementary Figures. [file 41598_2024_56851_MOESM1_ESM.docx]

**Appendix:**

Time history responses of FE models subjected to El-Centro horizontal excitation.

**Base Shear Responses**


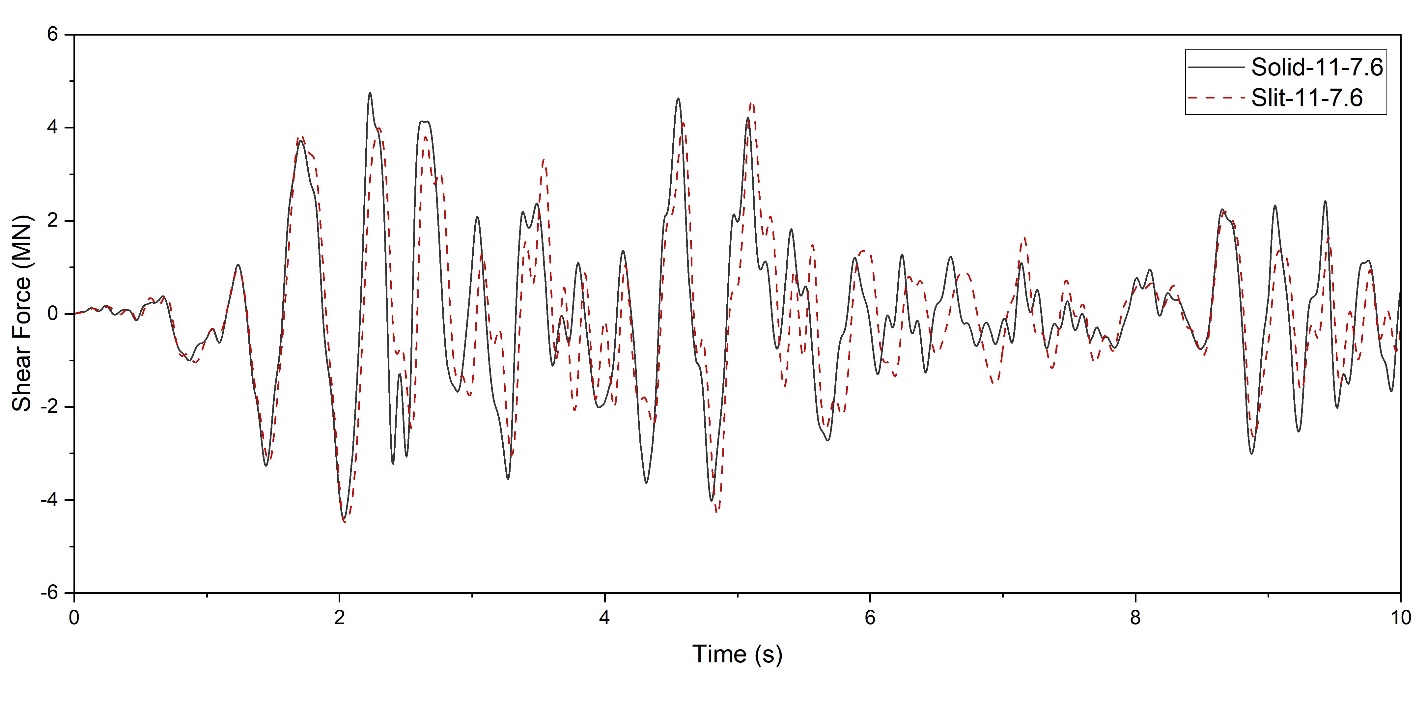


*Fig. 1. Time history base shear response of Solid-11-7.6 and Slit-11-7.6 FE models*


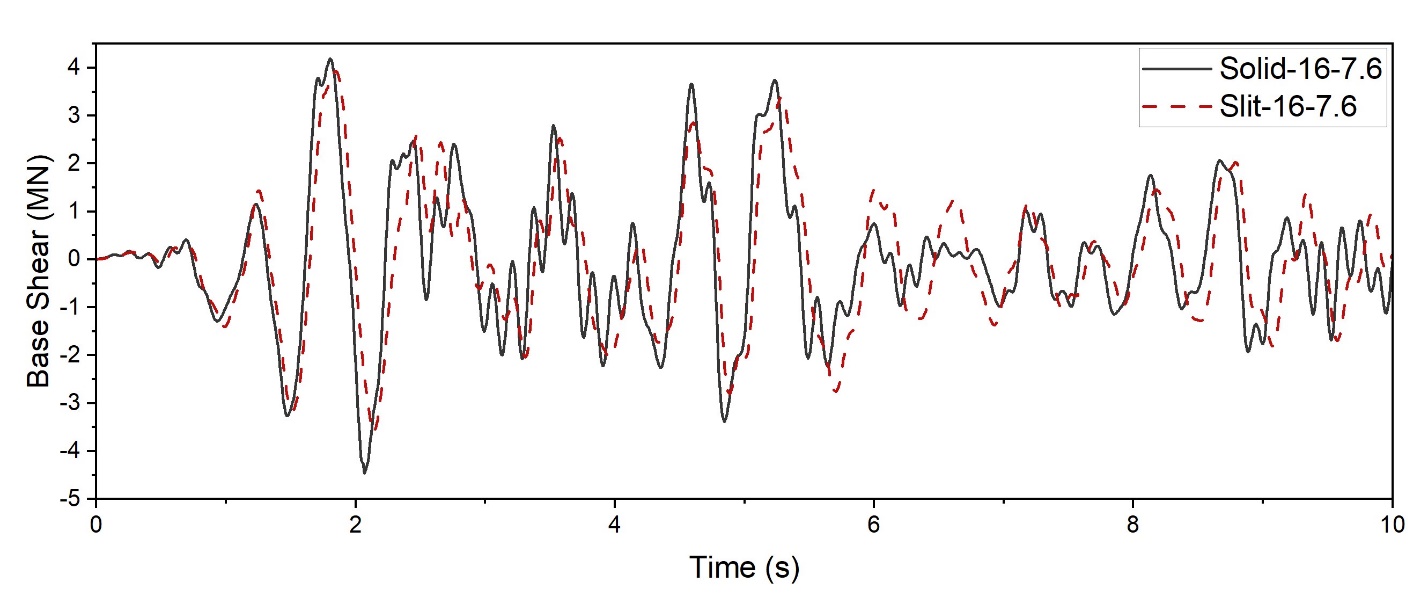


*Fig. 2. Time history base shear response of Solid-16-7.6 and Slit-16-7.6 FE models*


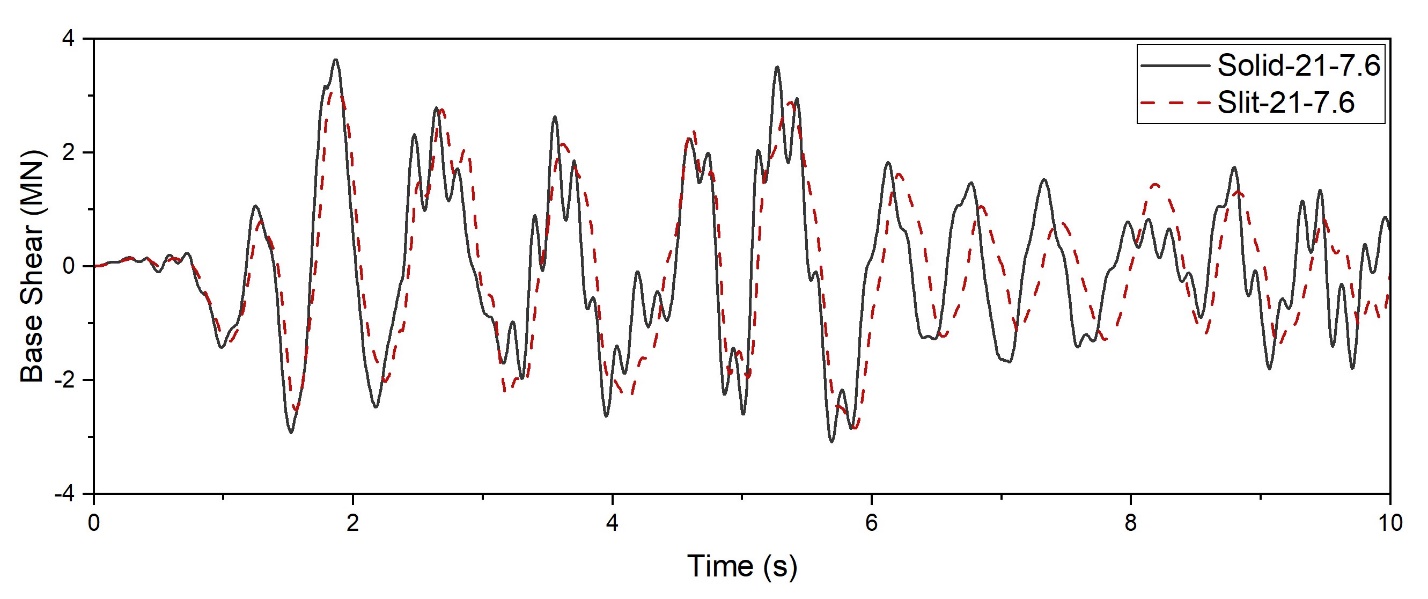


*Fig. 3. Time history base shear response of Solid-21-7.6 and Slit-21-7.6 FE models*


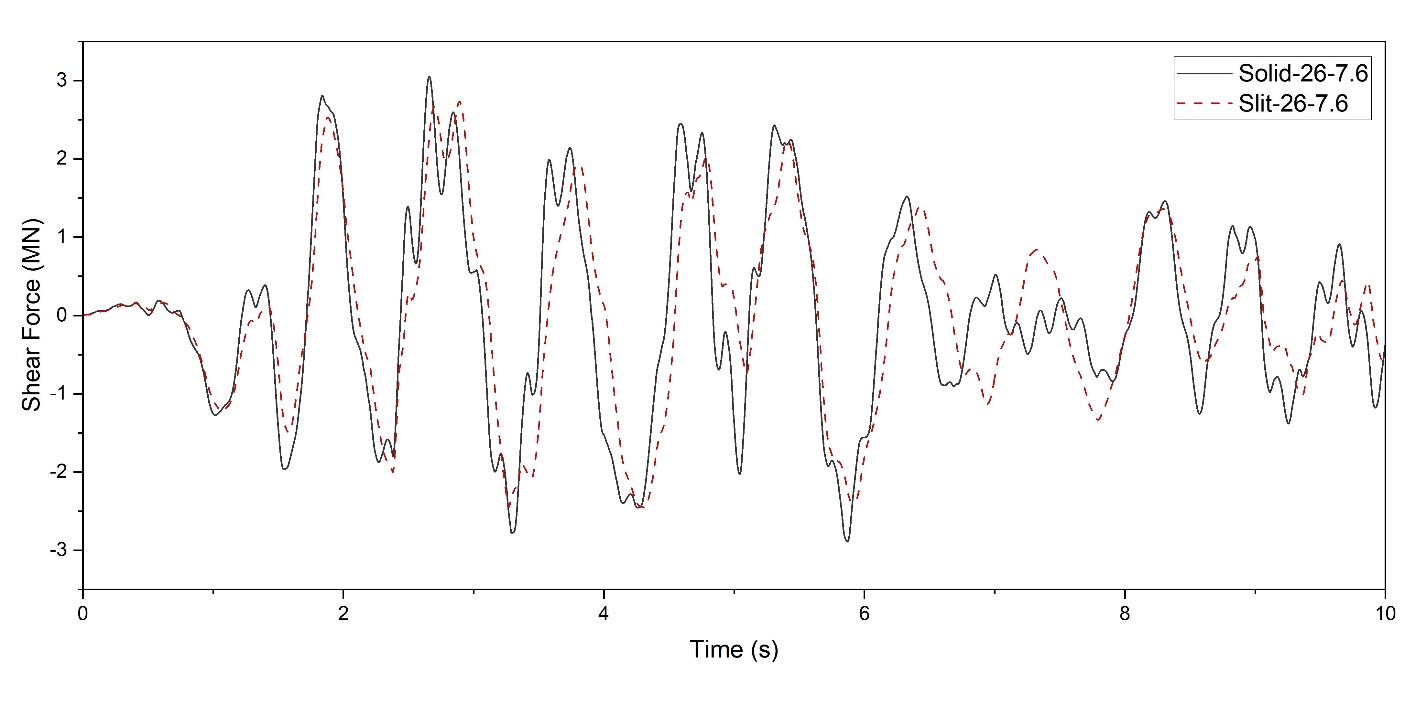


*Fig. 4. Time history base shear response of Solid-26-7.6 and Slit-26-7.6 FE models*


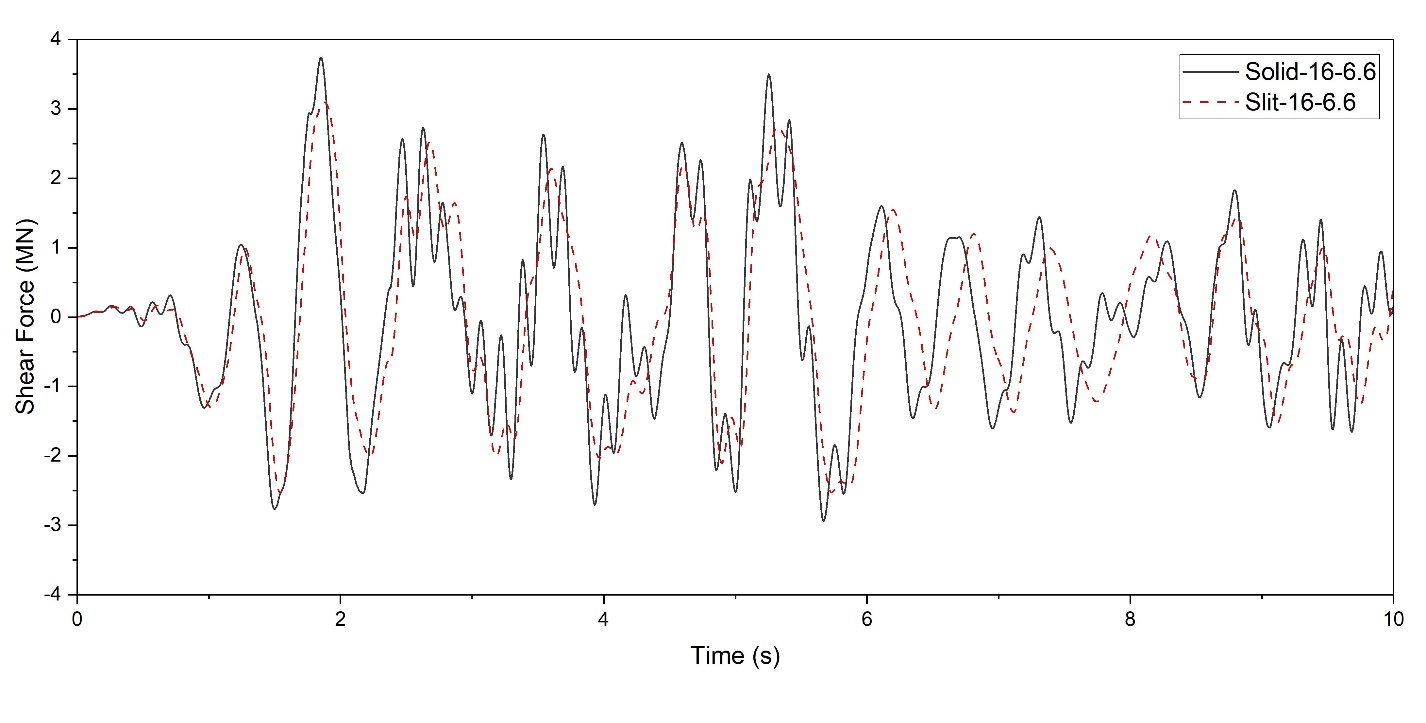


*Fig. 5. Time history base shear response of Solid-16-6.6 and Slit-16-6.6 FE models*


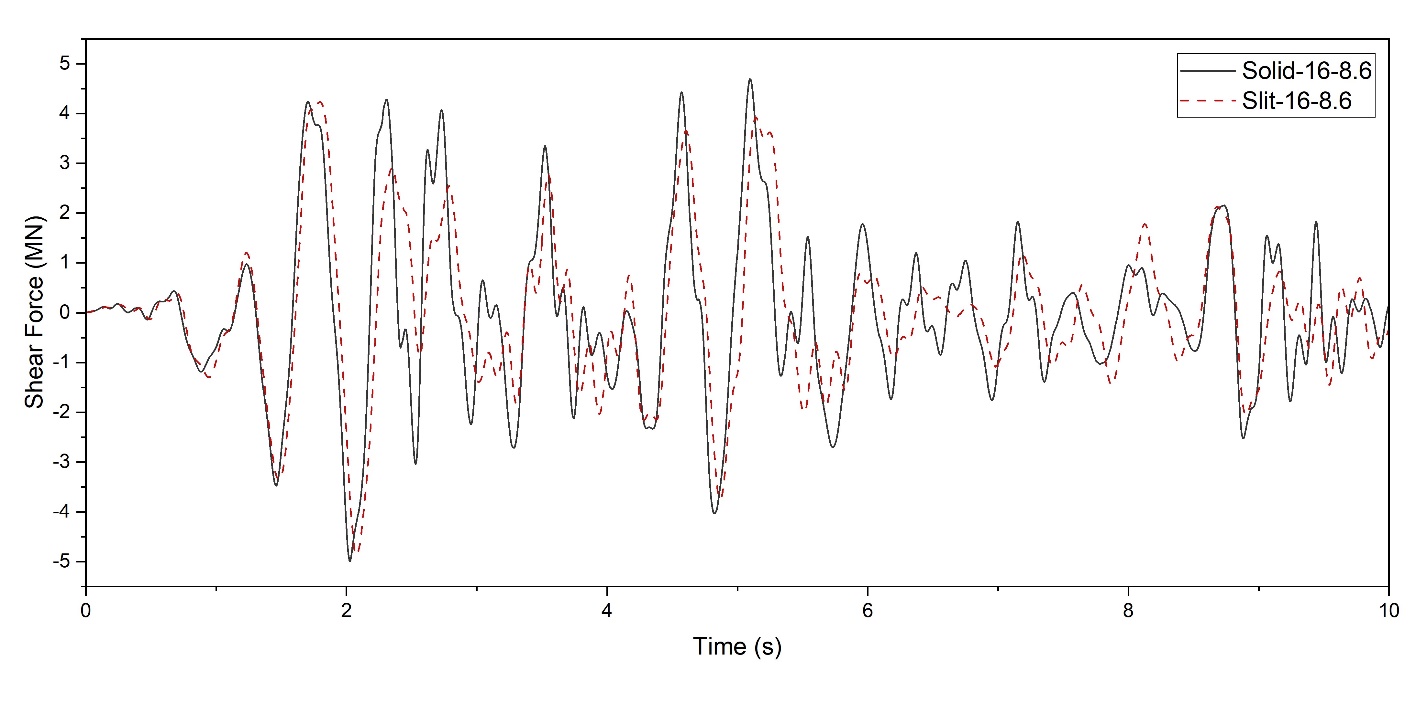


*Fig. 6. Time history base shear response of Solid-16-8.6 and Slit-16-8.6 FE models*

**Base Moment Responses**


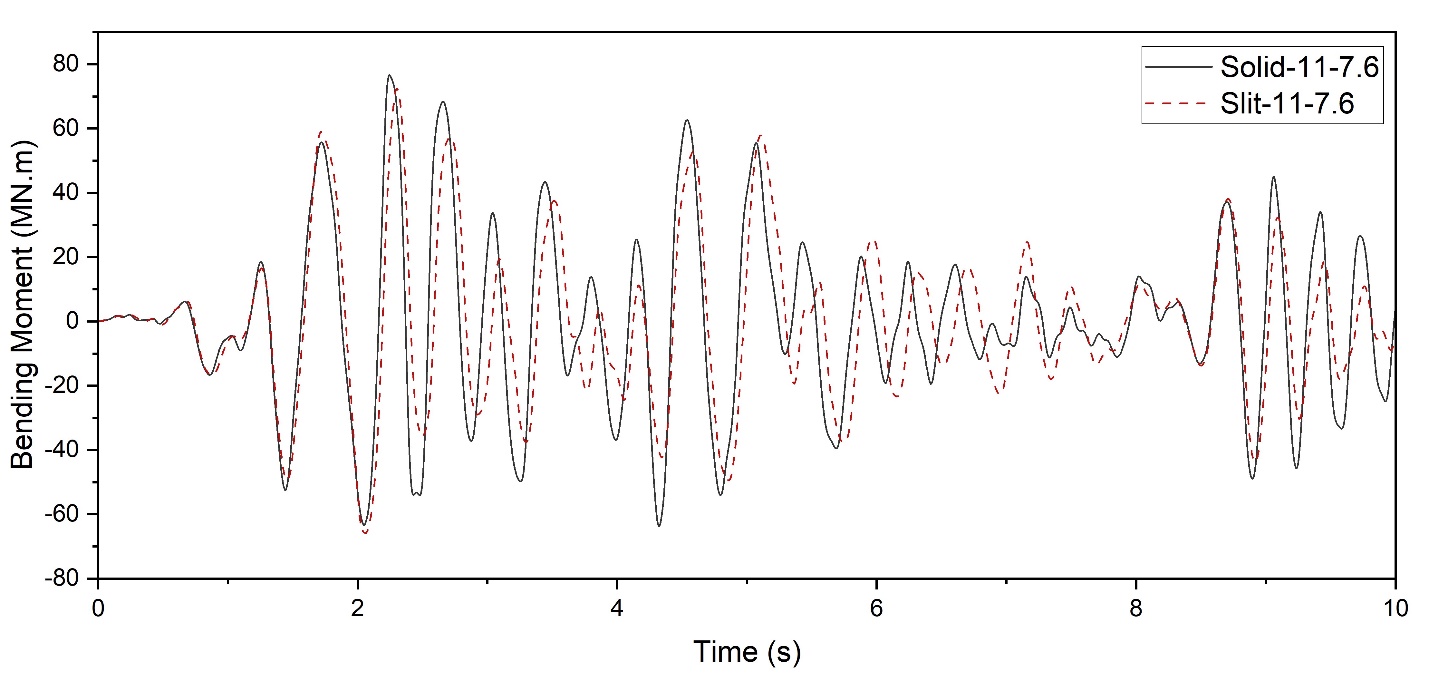


*Fig. 7. Time history Base Moment response of Solid-11-7.6 and Slit-11-7.6 FE models*


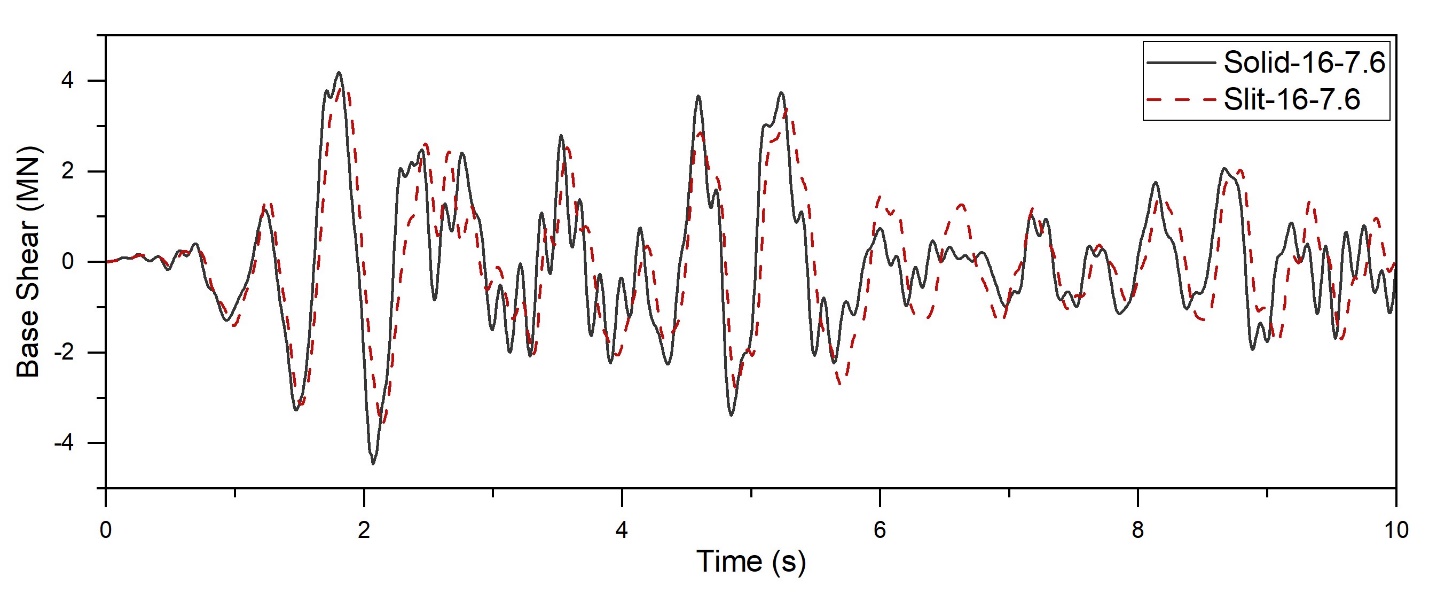


*Fig. 8. Time history Base Moment response of Solid-16-7.6 and Slit-16-7.6 FE models*


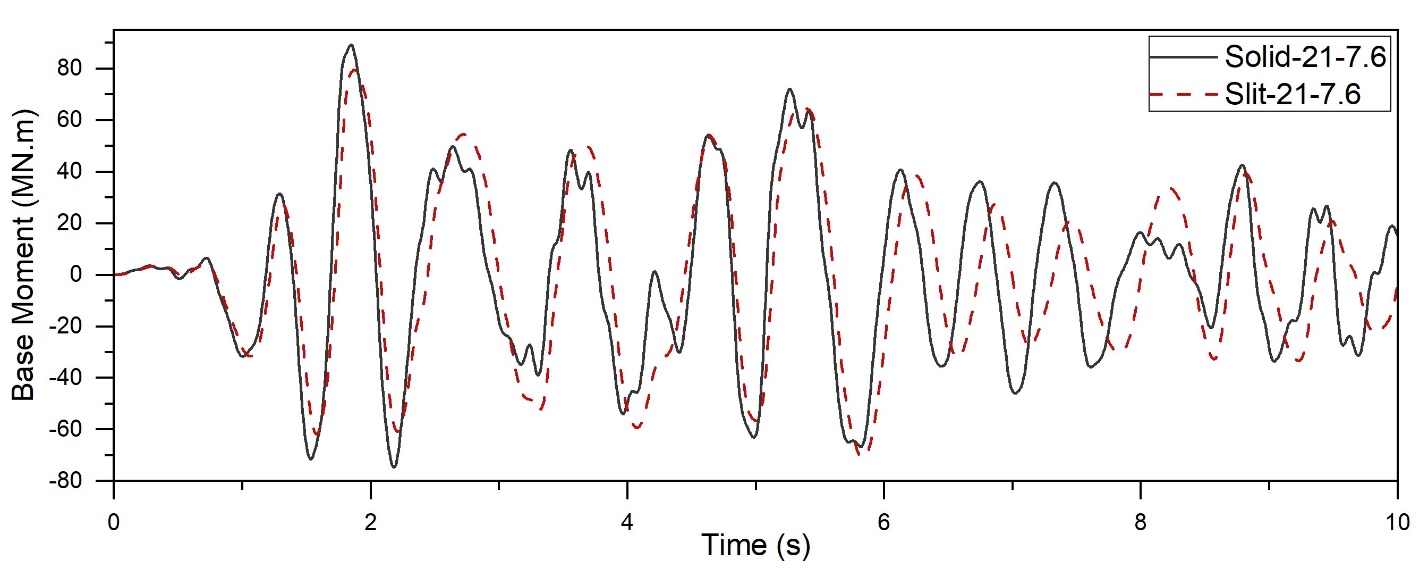


*Fig. 9. Time history Base Moment response of Solid-21-7.6 and Slit-21-7.6 FE models*


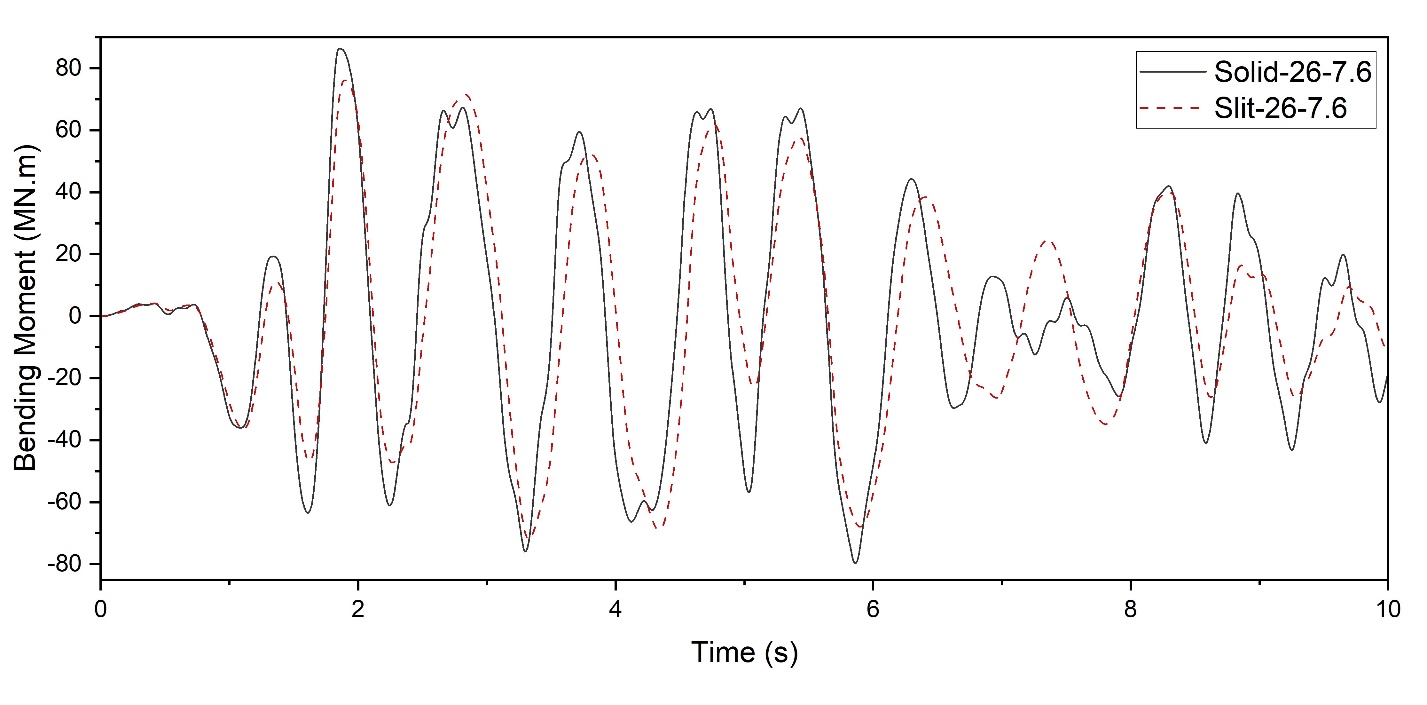


*Fig. 10. Time history Base Moment response of Solid-26-7.6 and Slit-26-7.6 FE models*


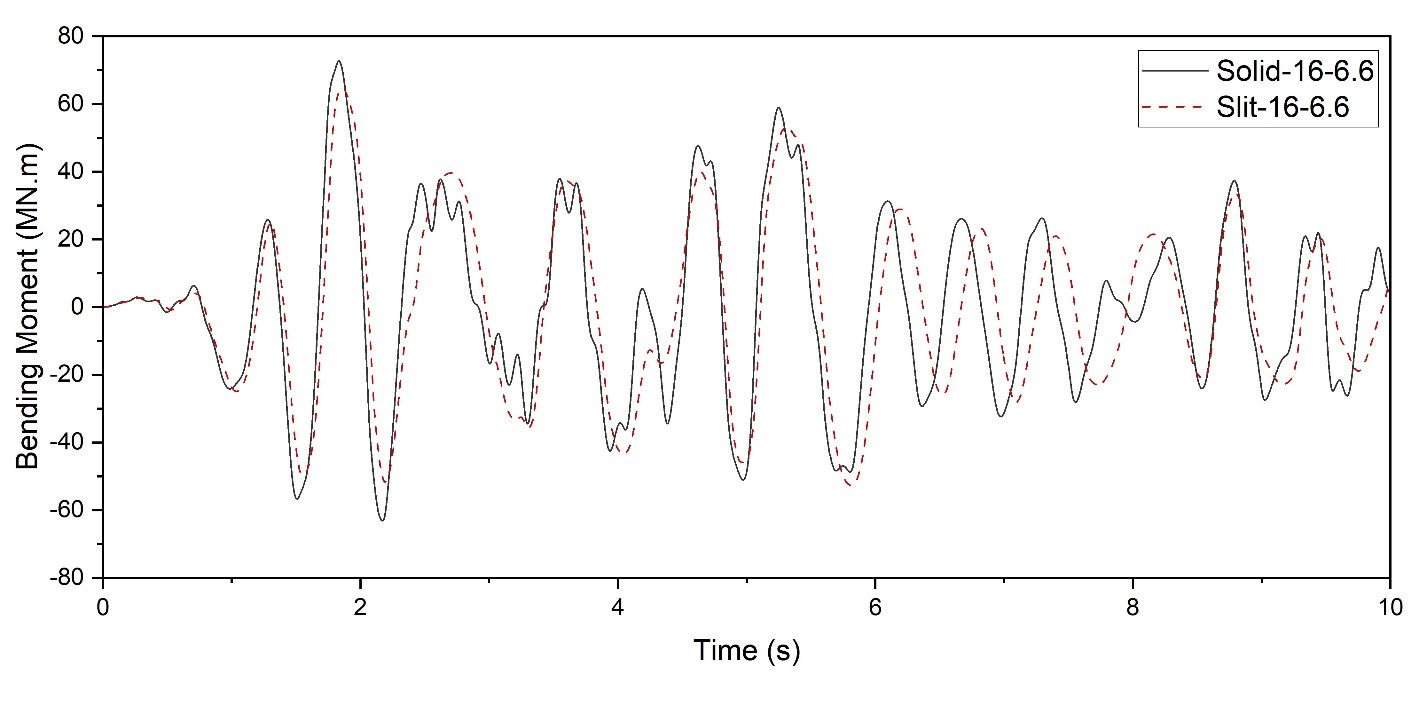


*Fig. 11. Time history Base Moment response of Solid-16-6.6 and Slit-16-6.6 FE models*


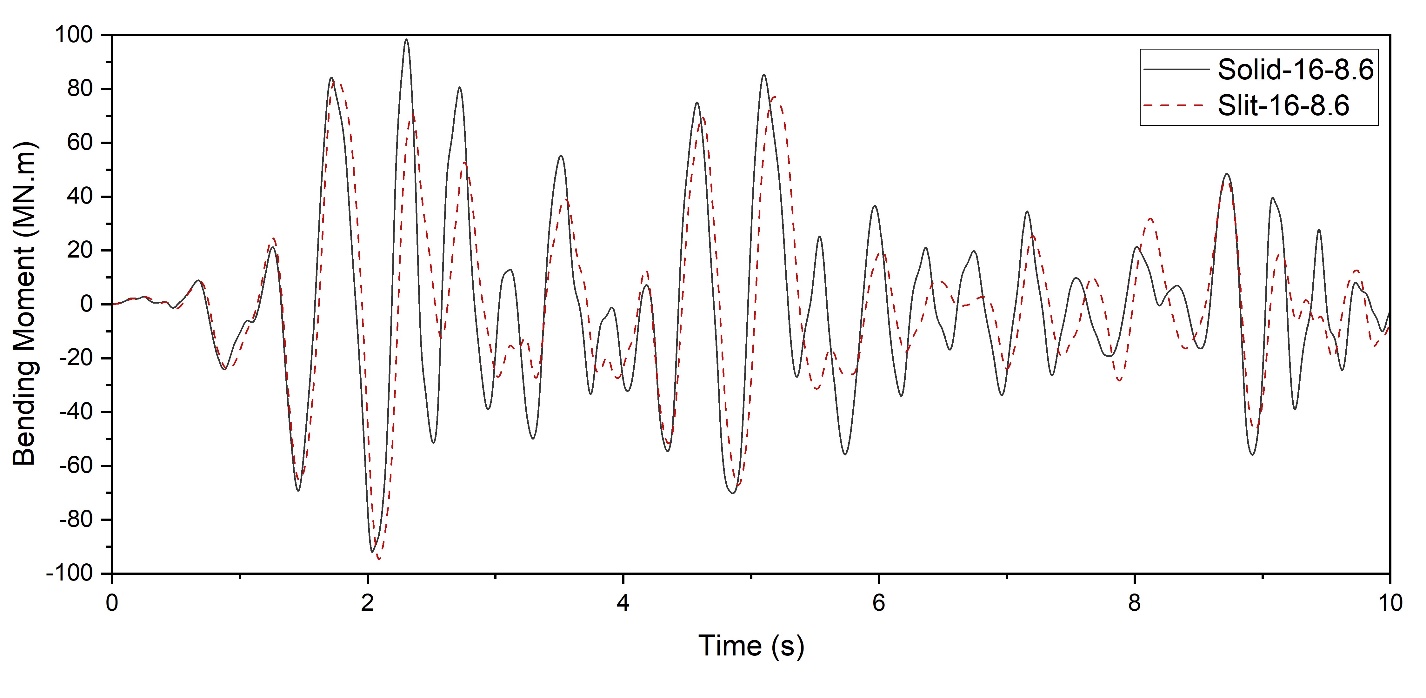


*Fig. 12. Time history Base Moment response of Solid-16-8.6 and Slit-16-8.6 FE models*

**Top Lateral Displacements**

**
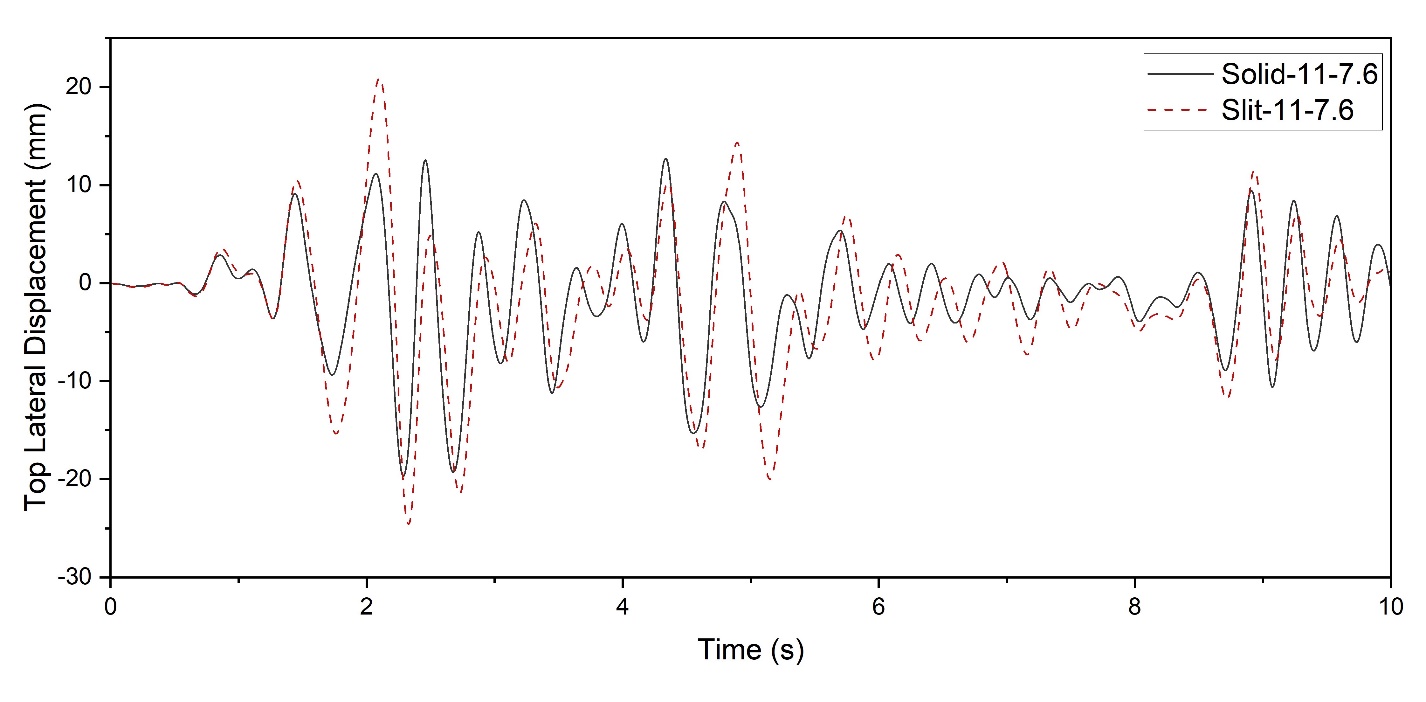
**

*Fig. 13. Time history Top Lateral Displacement response of Solid-11-7.6 and Slit-11-7.6 FE models*

**
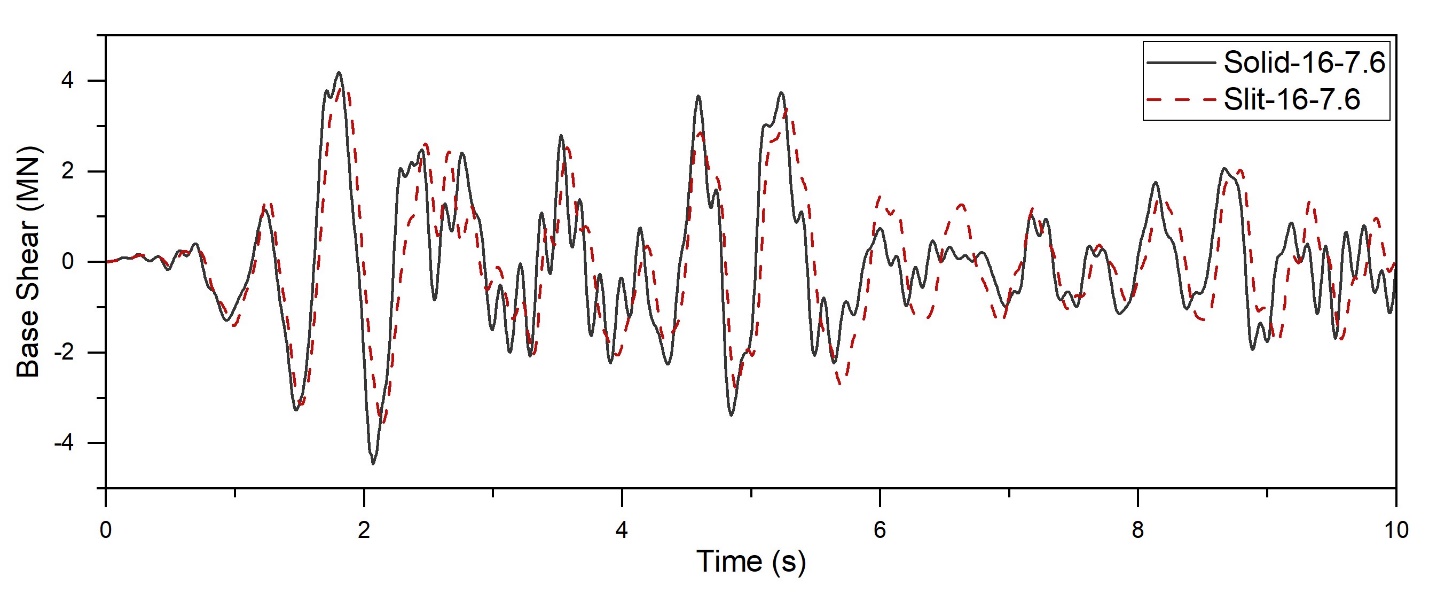
**

*Fig. 14. Time history Top Lateral Displacement response of Solid-16-7.6 and Slit-16-7.6 FE models*

**
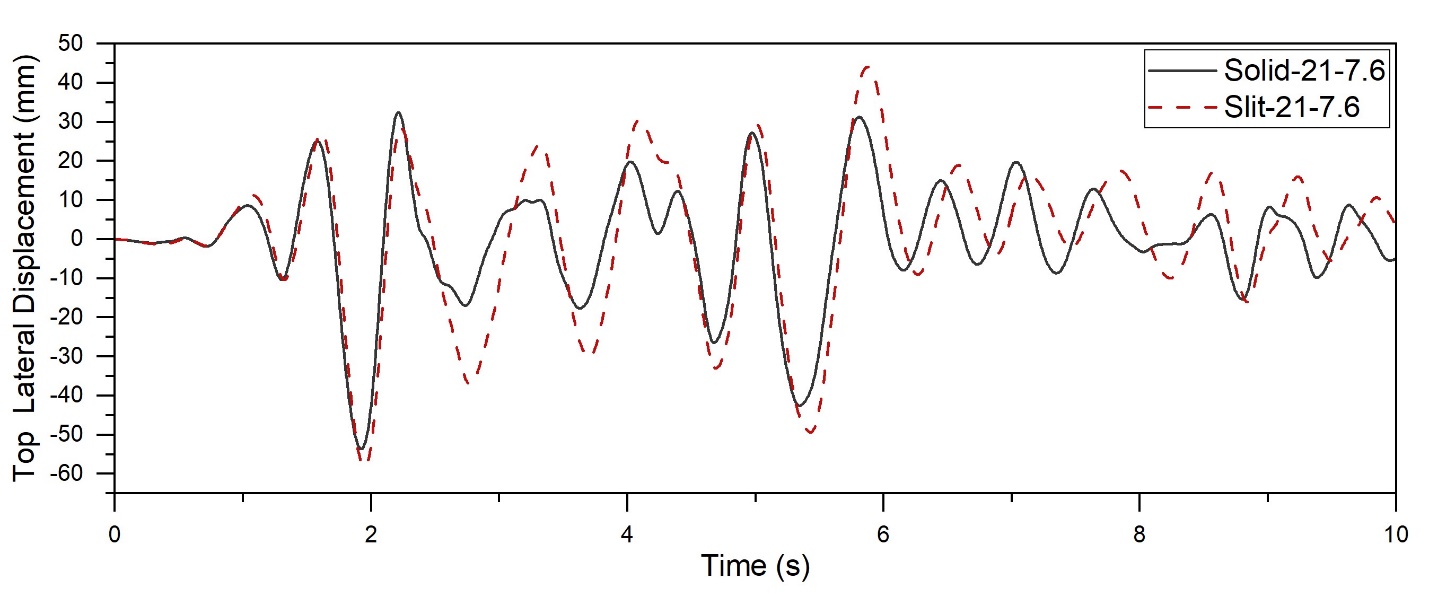
**

*Fig. 15. Time history Top Lateral Displacement response of Solid-21-7.6 and Slit-21-7.6 FE models*


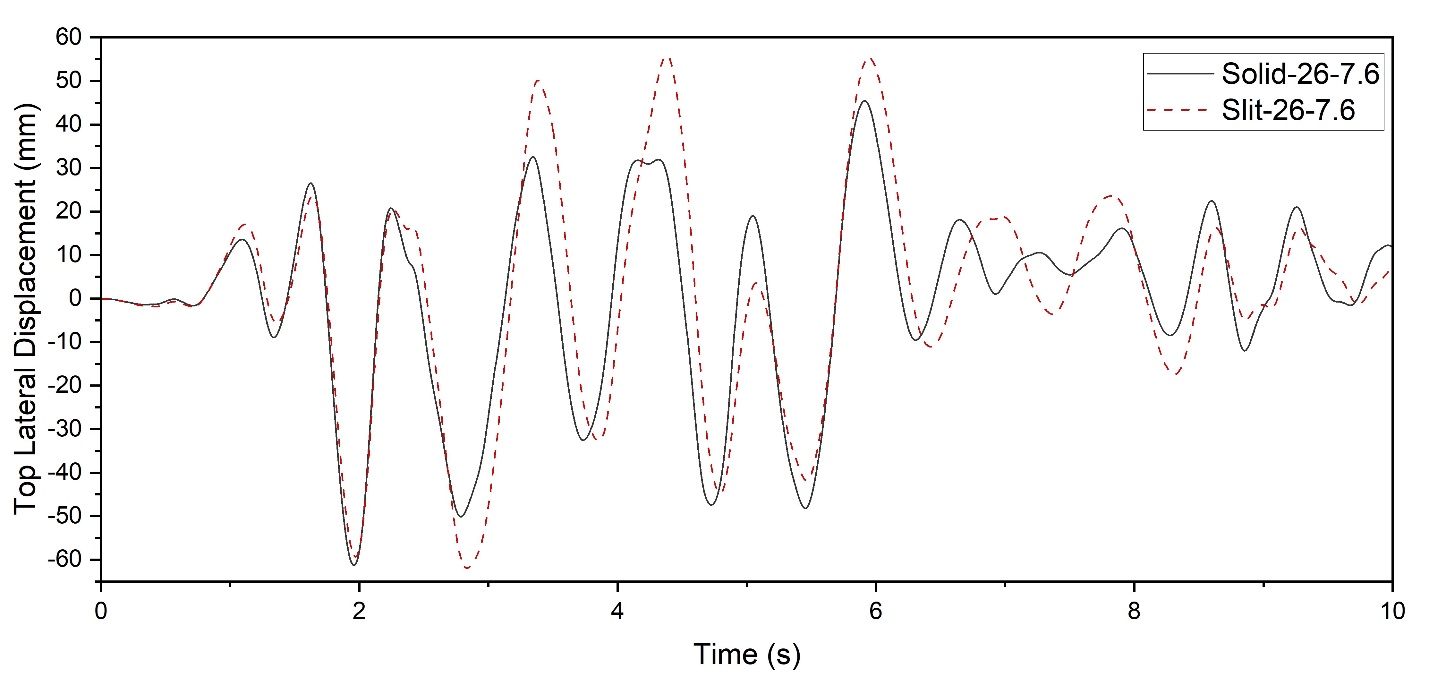


*Fig. 16. Time history Top Lateral Displacement response of Solid-26-7.6 and Slit-26-7.6 FE models*


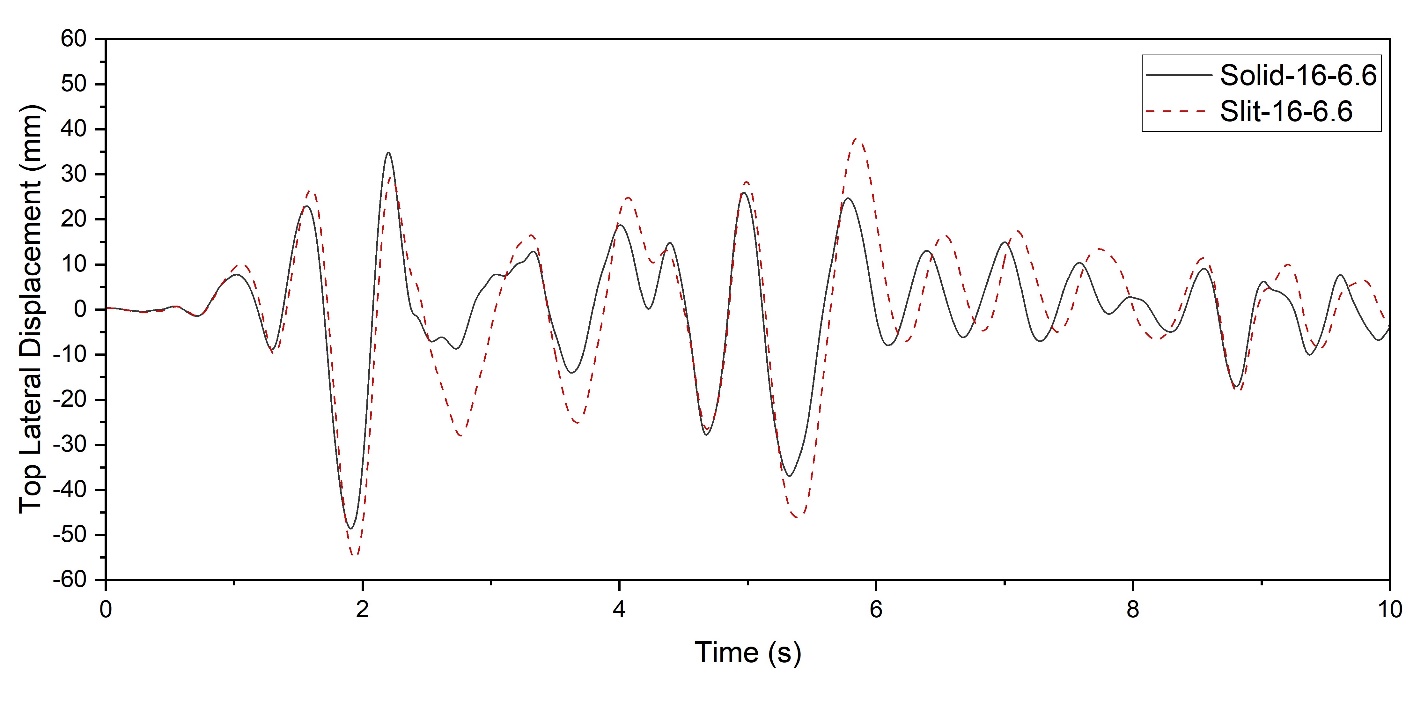


*Fig. 17. Time history Top Lateral Displacement response of Solid-16-6.6 and Slit-16-6.6 FE models*


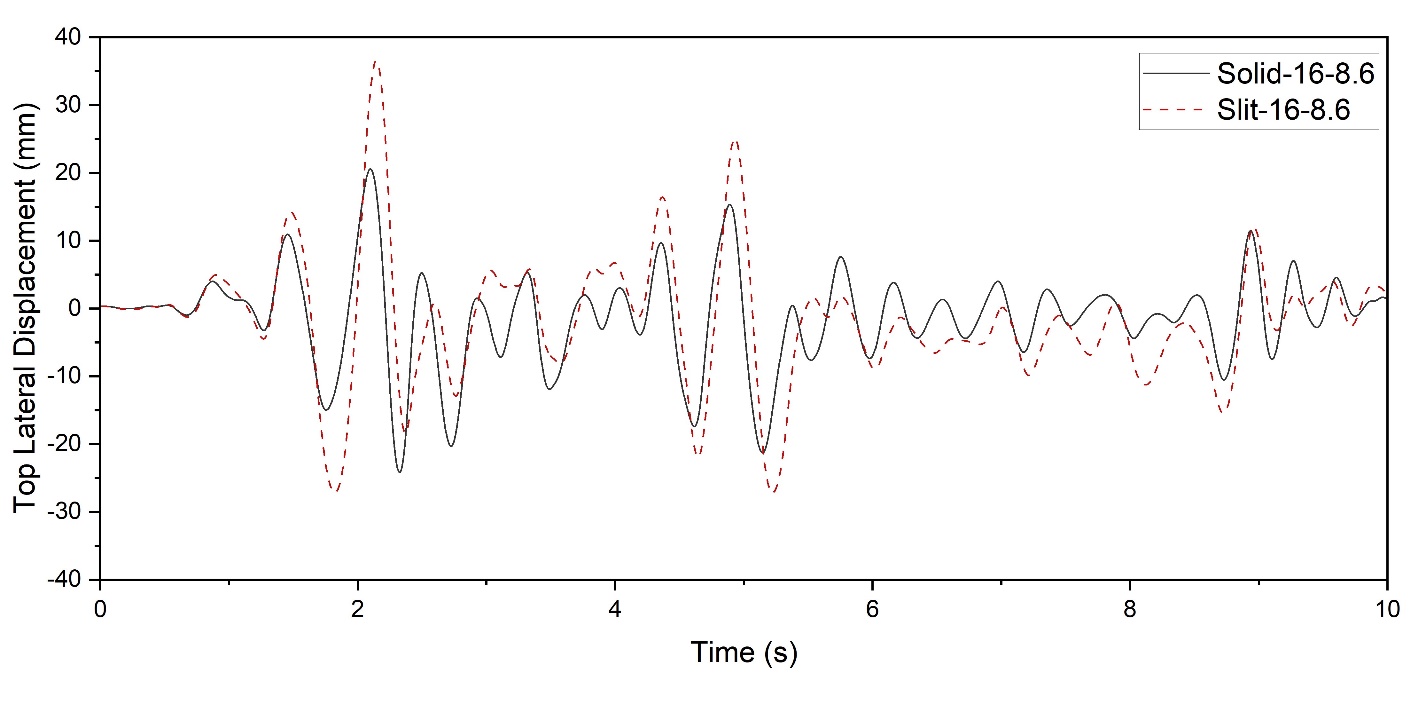


*Fig. 18. Time history Top Lateral Displacement response of Solid-16-8.6 and Slit-16-8.6 FE models*
